# Cold storage delays peach fruit softening via m^6^A reader PpYTHDFE1 liquid-liquid phase separation-mediated degradation of cell wall-loosening transcript *PpEXP3*

**DOI:** 10.1186/s43897-025-00218-3

**Published:** 2026-05-09

**Authors:** Hanqing Wang, Hanxiao Bian, Xubin Wu, Kunsong Chen, Donald Grierson, Bo Zhang

**Affiliations:** 1 Department of Horticulture, Colleague of Agriculture and Biotechnology, Zhejiang University, Hangzhou, 310058 China; 2 Zhejiang Key Laboratory of Horticultural Crop Quality Improvement, Zhejiang University, Hangzhou, 310058 China; 3 Plant Sciences Division, School of Biosciences, University of Nottingham, Sutton Bonington Campus, Loughborough, LE12 5RD UK; 4 Hainan Institute of Zhejiang University, Sanya, Hainan 572000 China

**Keywords:** Epitranscriptome, Transcriptome, Liquid–liquid phase separation, Low temperature, Fruit quality, Postharvest biology

## Abstract

N^6^-methyladenosine (m^6^A), the most prevalent epitranscriptomic modification in plant mRNAs, functions as a critical regulatory layer governing environmental response. Refrigeration reduces transcripts of cell wall enzymes to delay softening, however whether m^6^A participated in this progress remains unclear. Here, we identified a cold-inducible m^6^A reader PpYTHDFE1 as a regulator that stabilizes peach (*Prunus persica* L.) fruit firmness. Epitranscriptomic analysis revealed that firmness maintenance correlates with reduced levels of m^6^A-modified transcripts such as expansin *PpEXP3*, whose overexpressing accelerates fruit softening. Both in vitro RNA-EMSA and in vivo RIP-qPCR assays demonstrate that PpYTHDFE1 recognizes m^6^A-modified *PpEXP3* transcripts. Transcription inhibition assays further confirm that PpYTHDFE1 accelerates the degradation of *PpEXP3* through RNA–protein interaction rather than transcriptional modulation. Mechanistically, PpYTHDFE1 participates in liquid–liquid phase separation as evidenced by fluorescence recovery after photobleaching (FRAP) assays, facilitating *PpEXP3* degradation*.* Furthermore, overexpression of *PpYTHDFE1* decreases *PpEXP3* mRNA levels, while silencing *PpYTHDFE1* leads to *PpEXP3* transcript accumulation. A significantly negative correlation was observed between *PpYTHDFE1* transcripts and firmness during fruit cold storage. Fruit firmness is increased by 44% in peach and 45% in tomato upon *PpYTHDFE1* overexpressing. Our findings provide molecular insights into RNA modification-based strategies to reduce postharvest losses and develop cold-resilient horticultural crops.

**Supplementary Information:**

The online version contains supplementary material available at 10.1186/s43897-025-00218-3.

## Core

Cold exposure reduces transcripts of cell-wall loosening and ethylene synthesis-related enzymes, while activating CBF-dependent COR genes. These changes correlate with m^6^A modifications. The cold-induced m^6^A reader PpYTHDFE1 enhances peach firmness via liquid-liquid phase separation by selectively degrading *PpEXP3*, a cell wall-loosening protein. This work elucidates the molecular mechanisms underlying cold storage-mediated extension of fruit shelf life.

## Gene & accession numbers

The genes AtECT8 (AT1G79270), PpYTHDFE1 (Prupe.4G124900), PpEXP3 (Prupe.6G075100), PpACO1 (Prupe.3G209900), PpNAC1 (Prupe.4G187100) and PpCBF6 (Prupe.5G090000) were analyzed in this study. The sequencing data are available in the Genome Sequence Archive (Genomics, Proteomics & Bioinformatics 2021) in National Genomics Data Center (Nucleic Acids Res 2022), China National Center for Bioinformation/Beijing Institute of Genomics, Chinese Academy of Sciences (GSA: CRA024139) that are publicly accessible at https://ngdc.cncb.ac.cn/gsa.

## Introduction

N^6^-methyladenosine (m^6^A) is the most prevalent RNA methylation modification in eukaryotes and plays a key role in regulating RNA metabolism and stress responses (Jia et al. 2011; Liu et al. 2014; Pendleton et al. 2017; Zaccara et al. 2019; Xu et al. 2021; Boulias & Greer 2023; Mateos et al. 2024). This dynamic modification is reversibly regulated by m^6^A methyltransferases (“writers”) and demethylases (“erasers”) (Meyer & Jaffrey 2017; Balacco & Soller 2019). Central to m^6^A function are YTH domain-containing reader proteins, which recognize m^6^A marks and influence mRNA fate by regulating stability, splicing, nuclear export, and translation initiation (Arribas-Hernández et al. 2018; Wei et al. 2018; Guo et al. 2022; Bian et al. 2024; Cai et al. 2024).

The functions of m^6^A reader proteins have been well characterized in model plants, especially *Arabidopsis thaliana* and tomato (*Solanum lycopersicum*), where they regulate key processes such as development, flowering, stress responses, and fruit quality (Arribas-Hernández et al. 2018; Wei et al. 2018; Amara et al. 2023; Bian et al. 2024; Cai et al. 2024). Recent studies show that plant m^6^A readers use liquid–liquid phase separation (LLPS) to mediate RNA processing. In Arabidopsis, conserved C-terminal ECT proteins—including ECT1, ECT2, ECT8, and CPSF30-L—undergo phase separation under specific stimuli (Arribas-Hernández et al. 2018; Song et al. 2021; Lee et al. 2024). For example, salt stress induces ECT8 to sequester m^6^A-tagged RNAs into cytoplasmic condensates, accelerating transcript decay (Cai et al. 2024). In tomato, SlYTH2 enhances flavor during ripening by LLPS-dependent translational reprogramming (Bian et al. 2024). Beyond model species, YTH readers have been identified in economically important crops such as litchi (*Litchi chinensi*s) (Tang et al. 2022), citrus (*Citrus sinensis*) (Ouyang et al. 2019), and Rosaceae species (Wang et al. 2025), with apple MhYTP2 shown to regulate stress pathways (Wang et al. 2017a, b; Guo et al. 2022). However, functional validation and mechanistic understanding of m^6^A readers in non-model plants remain limited.

Low temperature is a key environmental factor affecting plant growth. The epigenetic modification m^6^A plays a role in plant responses to cold stress. In Arabidopsis, low temperature reduces m^6^A levels, triggering stress resistance mechanisms (Govindan et al. 2022; Wang et al. 2023). In tomato, cold-induced anther developmental defects are associated with decreased m^6^A levels (Yang et al. 2021). Knocking out the m^6^A writer MTA severely impairs Arabidopsis cold tolerance (Govindan et al. 2022; Wang et al. 2023). Similarly, downregulating FIP37 compromises photosynthetic efficiency under cold conditions, reducing cold tolerance (Vicente et al. 2023). The m^6^A eraser AtALKBH9C contributes to seed cold resistance; its knockout significantly lowers germination rate after cold stress (Amara et al. 2022). Despite growing evidence for m^6^A’s role in cold responses, the function of m^6^A readers remains largely unknown.

Low temperature poses challenges for plant growth but is essential for food safety and reducing food loss and waste (FLW), a global market failure resulting in over US$1 trillion worth of wasted food annually. While vast amounts of food are discarded, up to 783 million people suffer from hunger each year. In 2022, fruits and vegetables had the highest FLW rate at 45% (Hamish Forbes et al. 2024), with 48.9% occurring during postharvest handling and storage (Xue et al. 2021). Postharvest fruit softening, driven by cell wall modifications, reduces transport resilience, marketability, and increases susceptibility to mechanical damage and pathogens, contributing to FLW (Seymour et al. 2013). This process involves coordinated changes in cell wall structure, including pectin depolymerization, cellulose reorganization, and hydrolase activity (Brummell 2006; Airianah et al. 2016; Shi et al. 2021). Functional genomics has identified key cell wall-modifying enzymes in horticultural crops such as tomato (*Solanum lycopersicum*), apple (*Malus domestica*), strawberry (*Fragaria* × *ananassa*), and pepper (*Capsicum annuum*) (Shi et al. 2023). CRISPR knockout of tomato expansin (S*lEXP1*) and cellulase (*SlCEL2*) improves fruit firmness and extends shelf life by strengthening cell walls (Su et al. 2024). Similarly, dual knockout of polygalacturonase (*SlPG2a*) and pectate lyase (*SlPL*) significantly prolongs tomato shelf life (Ortega-Salazar et al. 2024). In the absence of genetic modification, cold storage remains the most effective method to delay softening and extend shelf life by slowing ethylene-mediated ripening and aging (Brizzolara et al. 2020).

*Prunus persica* L., an economically important fruit originating from China and now grown worldwide, is a major agricultural commodity with global production reaching 27.08 million metric tons across 1.56 million hectares (Food and Agriculture Organization, https://www.fao.org/faostat/). Postharvest softening severely reduces peach marketability by increasing susceptibility to bruising and pathogen decay, leading to significant economic losses. Although endo-polygalacturonases (endo-PGs) are associated with textural changes (Morgutti et al. 2006; Gu et al. 2016), the regulatory networks controlling cell wall remodeling remain poorly understood. Cold storage is currently the primary method for delaying postharvest deterioration (Brizzolara et al. 2020). Our previous work showed that DNA methylation, a key epigenetic modification, plays a role in the cold response of peach fruit and helps maintain firmness (Duan et al. 2023). However, the involvement of other epigenetic modifications, such as RNA methylation, in this process remains unclear.

In this study, we identify the peach YTH-domain protein PpYTHDFE1 as an m^6^A reader that regulates postharvest physiology by coupling RNA methylation decoding with phase separation. Through integrated transcriptomic, epigenomic, and LLPS analyses, we show that PpYTHDFE1 is highly expressed under cold conditions and undergoes phase separation. It promotes the degradation of m^6^A-modified *PpEXP3* transcripts, thereby preserving fruit firmness. These findings uncover a novel mechanism in which cold storage signals regulate cell wall metabolism via RNA modifications, offering potential for extending shelf life of perishable fruits through targeted modulation of m^6^A reader proteins.

## Results

### Cold storage maintains fruit firmness

**Fig. 1:**
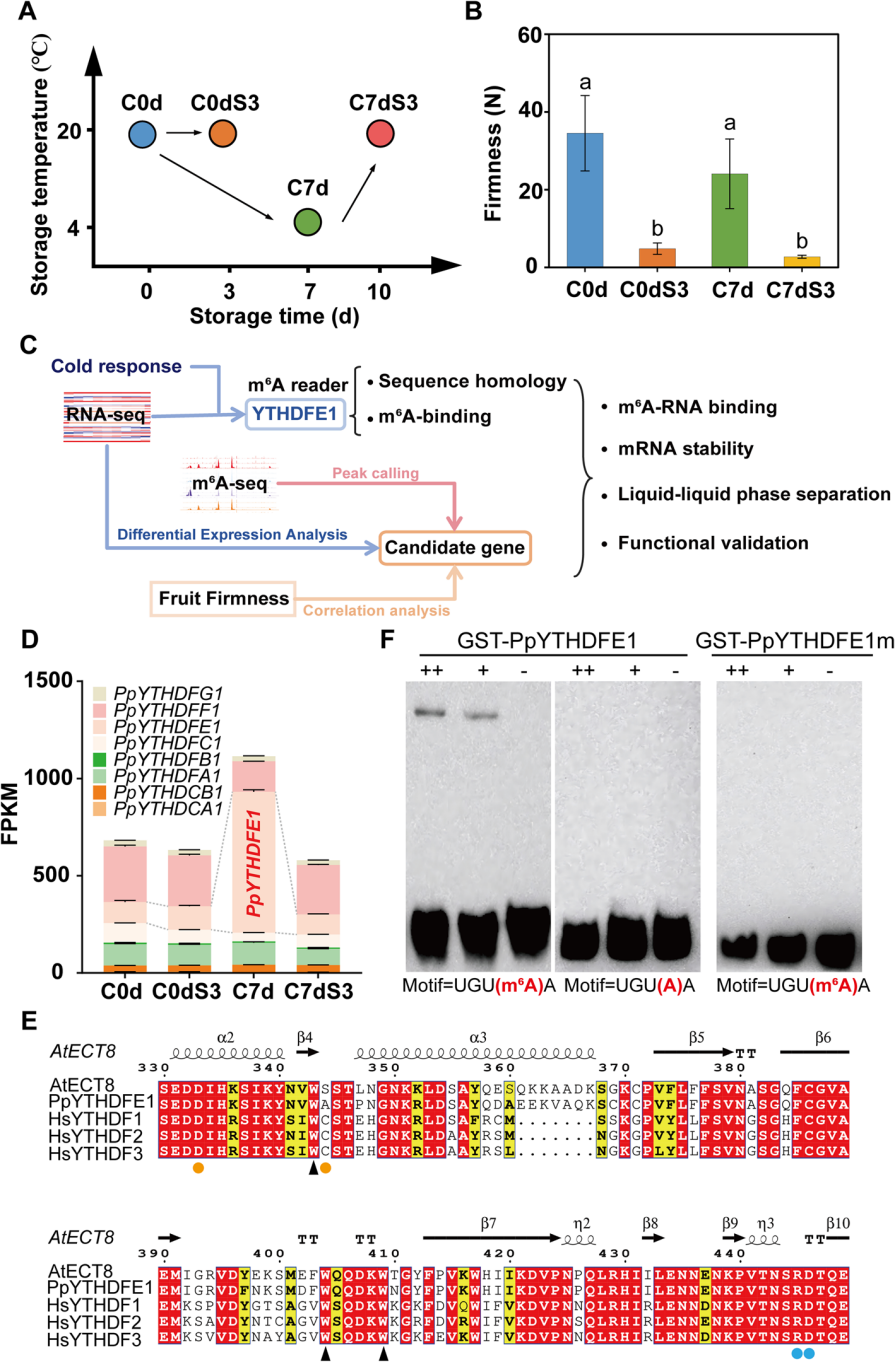
PpYTHDFE1 is an m^6^A reader induced by low-temperature storage. **A** Schematic diagram of peach fruit storage experiment. C0d, peach fruit at harvest; C0dS3, fruit stored at 20 ℃ for 3 days; C7d, fruit following 7 days of cold storage; C7dS3, fruit stored in the cold for 7 days followed by a 3 days recovery at 20 ℃. **B** Firmness changes during peach storage. Data are presented as mean ± SD of nine independent biological replicates. Significant differences (Tukey’s multiple range test, *P* < 0.05, F = 13.36) are denoted with different lowercases. **C** Summary of the research strategy in this study. **D** Expression profile of eight peach YTH genes during low temperature storage. Data are presented as mean ± SD of three independent biological replicates. The dashed line marks the color block representing *PpYTHDFE1*. FPKM, Fragments Per Kilobase per Million. **E** Multiple sequence alignment of PpYTHDFE1 with m^6^A readers in *Arabidopsis thaliana* (At) and *Homo sapiens* (Hs). The secondary structural elements of the YTH domain from AtECT8 are shown above. The black triangles indicate conserved tryptophan for aromatic cage. Yellow circle indicates residues that contact m^6^A. Blue circles indicate residues that contact RNA. **F** EMSA showing that GST-PpYTHDFE1 specifically binds m^6^A-modified UGUAA motif and the m^6^A-binding ability is abolished in GST-PpYTHDFE1m. Each channel was loaded with decreasing concentrations of protein and a consistent amount of RNA oligo with a final concentration of 10 nM

Consumers generally store fruits in the refrigerator following purchase to prevent spoilage and prolong shelf life. To investigate the biological mechanisms underlying the common practice of refrigerating fruits, we conducted a postharvest storage experiment. As shown in Fig. 1A, peach fruits were divided into four groups: C0d (the day of harvest), C0dS3 (stored at 20 ℃ for 3 days), C7d (stored at 4 ℃ for 7 days), and C7dS3 (stored at 4 ℃ for 7 days followed by 3 days at 20 ℃). Peach fruit firmness, initially ~ 36 N at C0d, significantly decreased to ~ 5 N after 3 days at 20 ℃ (C0dS3). Low temperature storage preserved firmness, maintaining ~ 24 N after 7 days (C7d). Rapid softening occurred upon transfer to 20 ℃ for 3 days (Fig. 1B). These results indicate that postharvest cold storage effectively maintains fruit firmness and facilitates subsequent softening during shelf-life storage.

### PpYTHDFE1 is a low-temperature induced m^6^A reader

To investigate the role of the m^6^A reader in regulating peach fruit firmness during cold storage, we integrated transcriptome based on RNA-sequencing (RNA-seq) and epitranscriptomic modifications derived from m^6^A-sequencing (m^6^A-seq) followed by gene function identification (Fig. 1C). Our previous research (Wang et al. 2025) identified eight YTH genes in peach, six of which belong to the YTHDF subfamily. Utilizing transcriptome data from both our current experiment (Fig. 1D) and earlier study (Fig. 1; Figure S1), we identified a YTHDF subfamily member, *PpYTHDFE1*, whose expression was significantly induced up to ~ sevenfold in response to low temperature. This elevated expression rapidly diminished upon return to room temperature (Fig. 1D).

PpYTHDFE1 is highly homologous to Arabidopsis ECT8, a known m^6^A reader crucial for environmental response (Wu et al. 2024; Cai et al. 2024). To explore whether peach cold-induced PpYTHDFE1 can recognize m^6^A modifications, we conducted a bioinformatic and biochemical analysis. Multiple sequence alignment using ESPript3 (Robert & Gouet 2014) showed highly conserved functional sites in PpYTHDFE1, AtECT8 and human YTHDFs (Fig. 1E), including the aromatic cage structure, m^6^A, and RNA contact sites. The 3D protein model predicted by AlphaFold2 (Tunyasuvunakool et al. 2021) revealed that PpYTHDFE1 and AtECT8 possess a similar aromatic cage structure primarily composed of tryptophan (Figure S2), with conserved tryptophan residues Trp-343, Trp-404 and Trp-409 in AtECT8, and Trp-358, Trp-419 and Trp-424 in PpYTHDFE1. Collectively, the conservation patterns from multiple alignments and protein structure models suggest that PpYTHDFE1 functions as an m^6^A reader.

We subsequently purified glutathione S-transferase (GST) tagged recombinant protein PpYTHDFE1 (GST-PpYTHDFE1) to perform electrophoretic mobility shift assays (EMSA) aimed at evaluating the m^6^A-modified RNA binding ability of PpYTHDFE1. To verify that the RNA binding activity of PpYTHDFE1 is dependent on m^6^A modification, we designed 5'-biotin-labeled RNA probes containing both m^6^A-modified and unmodified UGUAA motifs. The results demonstrate that GST-PpYTHDFE1 specifically binds to m^6^A-modified probes, but not to the unmodified probes. Furthermore, to elucidate the role of the aromatic cage structure in the YTH domain in m^6^A-binding, we purified a point-mutated protein, GST-PpYTHDFE1m (W451A), for EMSA analysis (Fig. 1F). This mutant protein failed to bind m^6^A-modified RNAs, confirming that the m^6^A-binding ability of PpYTHDFE1 relies on the integrity of the aromatic cage formed by conserved tryptophan residues. These findings provide in vitro evidence of PpYTHDFE1's m^6^A-binding capability and underscore the critical importance of conserved tryptophan residues in this process.

### Epitranscriptome and transcriptome changes in response to low temperature

Having established that PpYTHDFE1 acts as an m^6^A reader with expression significantly induced by low temperature, we conducted m^6^A-seq with three biological replicates to examine the m^6^A profiling during fruit cold storage. This generated between 33 to 55 million raw reads per library (Table S1). We identified 12,050, 12,108, 11,238, and 11,435 high-confidence m^6^A peaks at the C0d, C0dS3, C7d, and C7dS3 stages, respectively (Figure S3A). Parallel RNA-seq analysis estimated 0.6 to 0.7 m^6^A peaks per transcript in transcriptome (Table S2), levels comparable to those in Arabidopsis and strawberry (Wei et al. 2018; Bian et al. 2024; Zhou et al. 2019, 2021), indicating that m^6^A modification is a common feature of mRNA in peach.

The majority of m^6^A-containing transcripts (approximately 60%) exhibit a single m^6^A peak (Figure S3B). The proportion of transcripts with two or more m^6^A peaks decreases after 7 days of cold storage, while the proportion with a single m^6^A peak increased. Motif analysis of m^6^A peaks identified a UGUAY (Y representing A, U, G, or C) sequence motif (Figure S3C), which is consistent with findings in Arabidopsis and tomato (Wei et al. 2018; Bian et al. 2024; Zhou et al. 2019). An evaluation of m^6^A peak distribution across the transcriptome revealed a high enrichment in the 3' untranslated region (3'UTR) and around the stop codons (53.8% ~ 61.9%), aligning with previous observations in tomato and strawberry fruit (Zhou et al. 2021, 2019). Fruit stored at low temperature (C7d) displayed higher density of m^6^A in the 3'UTR and stop codon compared to other stages (Figure S3D).

To further investigate the role of m^6^A in responses to low temperature, we classified genes into two groups: m^6^A targeted and non-m^6^A targeted. By analyzing the expression changes of these genes, we observed that m^6^A consistently exerts a stabilizing effect on gene expression, maintaining the transcriptome in a relatively stable state (Figure S4).

**Fig. 2:**
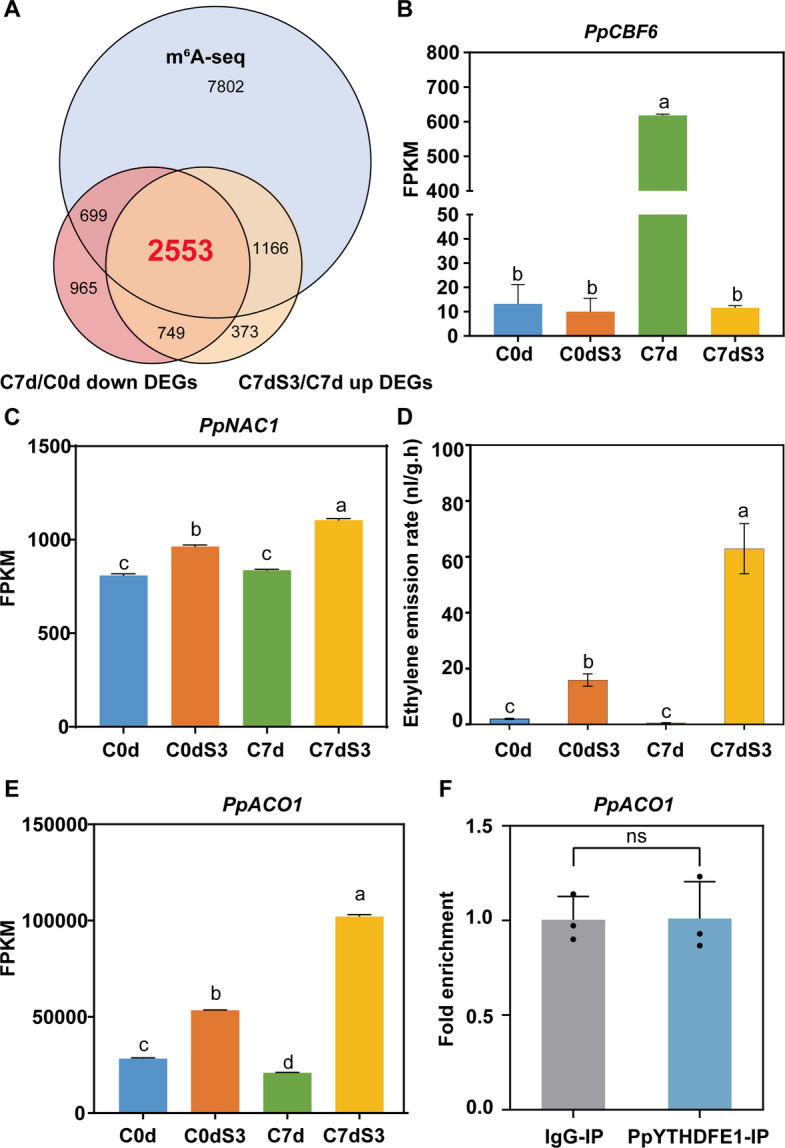
PpYTHDFE1 cannot directly affect ripening related genes. **A** Venn diagrams showing the overlap genes of DEGs decreased from C0d to C7d, DEGs increased from C7d to C7dS3 and m^6^A genes. **B** Expression of *PpCBF6* was significantly induced by cold storage. Data are presented as mean ± SD of three independent biological replicates. Significant differences (Tukey’s multiple range test, *P* < 0.05, F = 10,087) are denoted with different lowercases. FPKM, Fragments Per Kilobase per Million. **C** Expression profile of *PpNAC1* during cold storage. Data are presented as mean ± SD of three independent biological replicates. Significant differences (Tukey’s multiple range test, *P* < 0.05, F = 876.6) are denoted with different lowercases. **D** Ethylene emission rate of peach fruit during storage. Significant differences (Tukey’s multiple range test, *P* < 0.05, F = 122.5) are denoted with different lowercases. Data are presented as mean ± SD of three independent biological replicates. **E** Expression of *PpACO1* during cold storage. Data are presented as mean ± SD of three independent biological replicates. Significant differences (Tukey’s multiple range test, *P* < 0.05, F = 12,597) are denoted with different lowercases. **F** RIP­qPCR showing the binding affinity of PpYTHDFE1 to *PpACO1 *in vivo (ns, no significant; Student’s *t* test, t = 0.04021, df = 4). Data are shown as mean ± SD (*n* = 3, each biological replicate is marked with one dot)

We next analyzed the changes in the transcriptome, finding 4966 significantly less-abundant transcripts following cold storage, and 4841 significantly more abundant transcripts after removal from cold storage (Fig. 2A). Then, we identified a total of 2553 differentially expressed genes (DEGs) with m^6^A modifications in response to low temperature. GO enrichment analysis showed “mRNA modification”, “cold acclimation” and “plastid organization”, all of which are closely related to our central mechanism of post-transcriptional regulation during cold storage. Additionally, cell wall related terms such as “cell wall pectin biosynthetic process”, “cell wall pectin metabolic process”, “cell wall polysaccharide biosynthetic process” and “cell wall macromolecule biosynthetic process” were highly enriched (Tables S3, S4), suggesting that cell wall metabolism is highly active and modulated by m^6^A modification during cold storage.

In addition, we also screened gene transcripts that were activated by low temperature and then decreased when transferred to 20 ℃, finding 5331 significantly more abundant transcripts following cold storage, and 5459 significantly less-abundant transcripts after removal from cold storage (Figure S5). A total of 2198 DEGs with m^6^A modifications were identified activated by low temperature (Table S5). GO enrichment analysis showed “ubiquitin-protein transferase activity”, “aminoacyltransferase activity”, “acyltransferase activity” and “calcium-dependent protein kinase activity” were highly enriched (Tables S6). These results indicate the formation of a complex regulatory network associated with m^6^A modification during cold storage, integrating processes such as proteolysis (ubiquitination), protein and lipid modification, and calcium signaling.

### Transcriptional and hormonal mediators of low temperature response and ripening

The C-repeat Binding Factor (CBF) pathway-mediated cold signaling cascade represents an evolutionary conserved regulatory module across plant species (Xie et al. 2018; Mei et al. 2023). Genome-wide characterization identified six *CBF* genes in peach, with *PpCBF6* emerging as the dominant cold-responsive member (Duan et al. 2023). Here, we observed *PpCBF6* exhibits striking cold-inducibility, showing a 46-fold transcriptional induction after 7 days storage at 4 ℃ (Fig. 2B). Although *PpCBF6* transcripts lack m^6^A modification (Table S7), analysis of the peach homologs of Cold-Regulated Genes (COR), the CBF regulon, revealed that 46.7% (49/105) COR genes possess m^6^A modifications (Table S7), indicating a regulatory network for CBF-m^6^A parallel regulation of downstream cold responsive genes.

We next systematically examined the expression dynamics and m^6^A profile of transcription factors (TFs) annotated in the Plant Transcription Factor Database (Jin et al. 2017) (Table S8). Quantitative profiling revealed that 39.1% (587/1500) of detected TFs demonstrate m^6^A modifications, suggesting extensive post-transcriptional regulation of TFs in response to low temperature. Notably, we identified that TF *PpMADS2* (Prupe.5G208400), previously implicated in fruit chilling response (Duan et al. 2023), undergoes m^6^A modification accompanied by transcriptional suppression under low temperature. Comparative analysis identified *PpNAC1* (Prupe.4G187100), a homolog of tomato ripening regulator *SlNOR* (Cao et al. 2024a), as displaying an increased expression pattern during shelf-life at 20 ℃ (Fig. 2C), yet it lacked detectable m^6^A modification (Table S8). These contrasting epigenetic regulatory patterns between *PpMADS2* and *PpNAC1* TFs highlights distinct molecular mechanisms governing gene expression during postharvest cold storage.

Ethylene plays a central regulatory role in regulating peach fruit ripening, including changes in quality attributes, such as fruit softening (Wang et al. 2017c; Cao et al. 2024a). Our data demonstrate that during cold storage of peach fruit, as firmness is maintained, the ethylene production correspondingly decreases (Fig. 2D). Previous studies have identified *PpACS1* and *PpACO1* as the key genes involved in ethylene biosynthesis (De Los Cobos et al. 2024), and the expression of these two genes is suppressed by low temperature (Fig. 2E; Table S9), confirming the results of previous study (Wang et al. 2017c). Notably, while m^6^A modification was detected on *PpACO1* transcripts (Figure S6), the m^6^A reader PpYTHDFE1 showed no binding affinity for these modified mRNAs (Fig. 2F). Crucially, although ethylene application accelerates fruit softening (Cao et al. 2024a), it fails to induce significant changes in *PpYTHDFE1* expression (Figure S7). This observation is further supported by the lack of *PpYTHDFE1* suppression following treatment with 1-methylcyclopropene (1-MCP), a well-known ethylene signaling inhibitor. These collective findings establish that PpYTHDFE1 operates independently of the ethylene biosynthesis pathway, while ethylene signaling does not modulate *PpYTHDFE1* expression. Furthermore, the expression patterns and m^6^A modification profiles of transcripts associated with abscisic acid (ABA), auxin (IAA) and jasmonic acid (JA) synthesis and signaling were also analyzed and identified (Table S9). This showed a complex m^6^A mediated transcriptional and hormone mediated regulatory network.

### Identification of cell wall genes with m^6^A modified transcripts

**Fig. 3:**
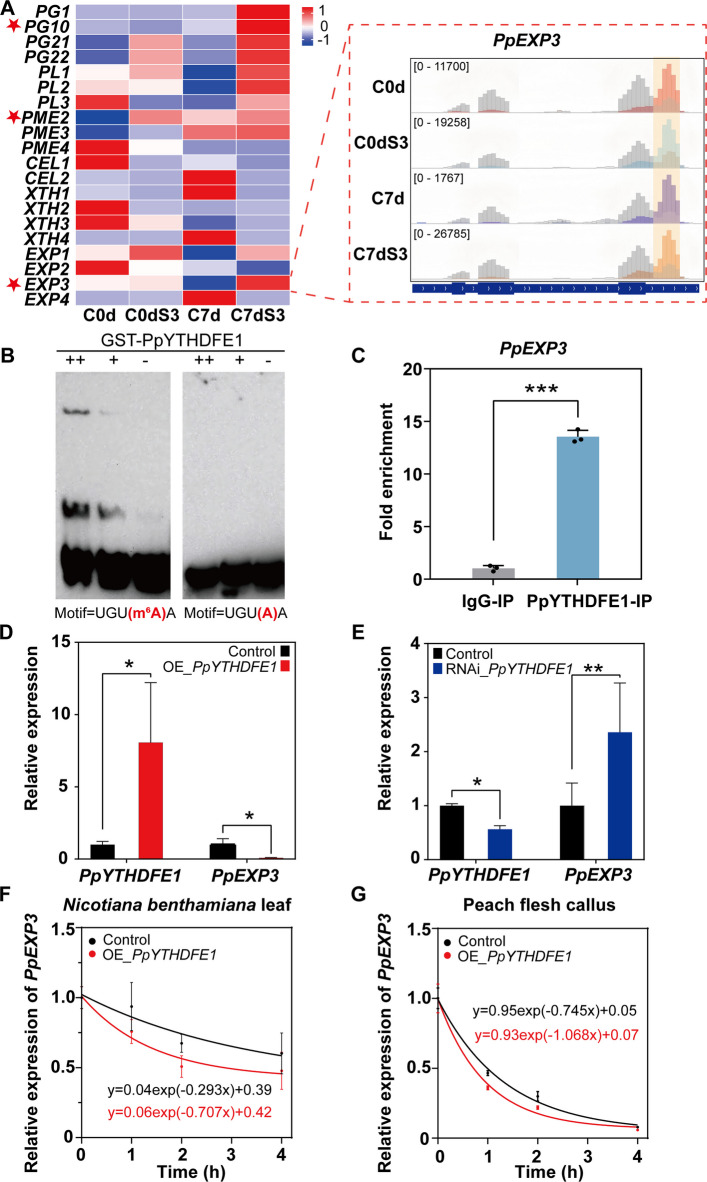
PpYTHDFE1 binds to m^6^A-*PpEXP3* mRNA and accelerates its degradation. **A** Expression profile and m^6^A methylation status of cell wall genes. Data are presented as the mean of three independent biological replicates. Heat map ranging from blue to red, reflecting normalized FPKM intensity. The genes with m^6^A modification are marked with a red star. m^6^A methylation status of *PpEXP3* during storage were shown by Integrative Genomics Viewer (IGV). The input reads are presented in the foreground. The yellow rectangle indicates the position of m^6^A targeted sites at 3' Untranslated Region (3’UTR). **B** EMSA showing that GST-PpYTHDFE1 specifically binds m^6^A-modified *PpEXP3*. Each channel was loaded with decreasing concentrations of protein and a consistent amount of RNA oligo with a final concentration of 10 nM. **C** RIP­qPCR showing the binding affinity of PpYTHDFE1 to *PpEXP3 *in vivo (***, *P* < 0.001; Student’s *t* test, t = 32.20, df = 4). Data are shown as mean ± SD (*n* = 3, each biological replicate is marked with one dot). **D** and **E** show the expression of *PpYTHDFE1* and *PpEXP3* in peach flesh callus transient overexpressing (**D**) and RNA interference (RNAi) (E) PpYTHDFE1, demonstrating its negative regulatory effect on *PpEXP3* expression (*, *P* < 0.05, **, *P* < 0.01; Student’s *t* test). Empty vectors serve as control. Data are presented as mean ± SD of three independent biological replicates. **F** and **G** show the RNA stability experiment results based on *Nicotiana benthamiana* leaf (**F**) and peach fruit callus tissue (**G**) overexpressing *PpYTHDFE1* transiently indicating PpYTHDFE1 can accelerate the degradation of *PpEXP3* mRNA. Data are presented as mean ± SD (*n* = 3 biological replicates)

To delineate the epitranscriptomic regulation of fruit texture, we conducted systematic analysis of transcripts encoding enzymes involved in fruit softening among 2553 intersecting DEGs (Figs. 2A, 3A) (Shi et al. 2023). Three cell wall genes were identified that exhibited m^6^A peaks (Fig. 3A), pectin methylesterase *PpPME2* (Prupe.2G279800), endo-polygalacturonase *PpPG10* (Prupe.2G014800) and expansin *PpEXP3* (Prupe.6G075100). Transcripts of *PpPME2* and *PpPG10* either showed no correlation with fruit firmness or the expression level was relatively low (FPKM < 10). Therefore, the expansin gene *PpEXP3* transcripts were selected as the only notable cold-responsive m^6^A target with conserved methylation at four storage stages. Quantification of transcriptional dynamics showed *PpEXP3* expression was suppressed by ninefold during cold storage at 4 ℃, but rapidly increases upon transfer to 20 ℃ (Table S10; Fig. 3A). This dramatic change in expression profile demonstrated a strong negative correlation with *PpYTHDFE1* accumulation (*r* = −0.829, *P* < 0.01, Figure S8), and was consistent with a role for *PpEXP3* in peach fruit softening (De Los Cobos et al. 2024).

In contrast, no m^6^A modifications were observed for endo-polygalacturonase (PG), such as *PpPG21* (Prupe.4G261900) and *PpPG22* (Prupe.4G262200), which are associated with peach fruit softening (Table S10) (Qian et al. 2021). Additionally, unlike *PpEXP3*, the expression of *PpPG21* and *PpPG22* increased after low temperature storage, revealing divergent regulatory mechanisms for different cell wall genes.

### PpYTHDFE1 recognizes m^6^A modified *PpEXP3* transcripts

We employed a biotin-labeled RNA probe targeting the 3’UTR region of *PpEXP3* to establish that PpYTHDFE1 directly regulates fruit firmness maintenance during cold storage through binding to and posttranscriptionally regulating the *PpEXP3* mRNA. RNA-EMSA results demonstrated that m^6^A-modified *PpEXP3* probes were recognized by GST-PpYTHDFE1 protein and binding increased as the concentration of the protein in the reaction mixture was raised. When m^6^A residues within the probes were substituted with adenine (A), GST-PpYTHDFE1 failed to recognize the RNA probes and binding was abolished (Fig. 3B), suggesting that the protein-RNA interaction is dependent on m^6^A modification.

To establish functional relevance, we performed RIP-qPCR on peach fruits using a polyclonal antibody specific to PpYTHDFE1 to immunoprecipitate the protein along with its associated RNAs. The RIP-qPCR assays revealed approximately 13-fold enrichment of *PpEXP3* transcripts in immunoprecipitated complexes compared to IgG (Fig. 3C, *P*< 0.001). This in vivo validation, combined with our biochemical evidence in vitro (Fig. 3B), conclusively demonstrates that PpYTHDFE1 recognizes m^6^A-epitranscriptomic marks on *PpEXP3* mRNA.

### PpYTHDFE1 accelerates the degradation of *PpEXP3* mRNA

Our findings established the m^6^A-dependent recognition of *PpEXP3* transcripts by PpYTHDFE1. In the absence of a tractable transgenic peach system, we developed a homologous transient expression system using clonal callus tissue from peach flesh. Crucially, these lines preserve native *PpYTHDFE1* induction under cold treatment (4 ℃), exhibiting threefold transcriptional activation (Figure S9), thus providing physiological relevance for functional studies.

Modulating *PpYTHDFE1* expression through *Agrobacterium*-mediated transient overexpression (eightfold induction) caused tenfold suppression of *PpEXP3* transcripts (Fig. 3D). Conversely, RNA interference (RNAi)-mediated knockdown (52% reduction) resulted in twofold increase in *PpEXP3* transcripts (Fig. 3E). This reciprocal regulation was corroborated by strong inverse correlation in transcriptomes of cold-stored fruits (*P* < 0.001, Figure S8), confirming PpYTHDFE1 as a posttranscriptional negative regulatory element of *PpEXP3* transcripts.

We conducted transcription inhibition assays under translation-independent conditions, in order to understand more about the mechanism of PpYTHDFE1 action. In heterologous (*Nicotiana benthamiana*) (Fig. 3F) and homologous (peach flesh callus) (Fig. 3G) systems, PpYTHDFE1-overexpressing lines showed approximately 58% and 30% reduction, respectively, in the half-life of *PpEXP3* transcripts following actinomycin D treatment. This epistatic relationship demonstrates that PpYTHDFE1 accelerates m^6^A-marked *PpEXP3* degradation through direct RNA–protein interaction rather than transcriptional modulation.

### Phase separation capacity of PpYTHDFE1 mediates m^6^A-dependent regulation

**Fig. 4:**
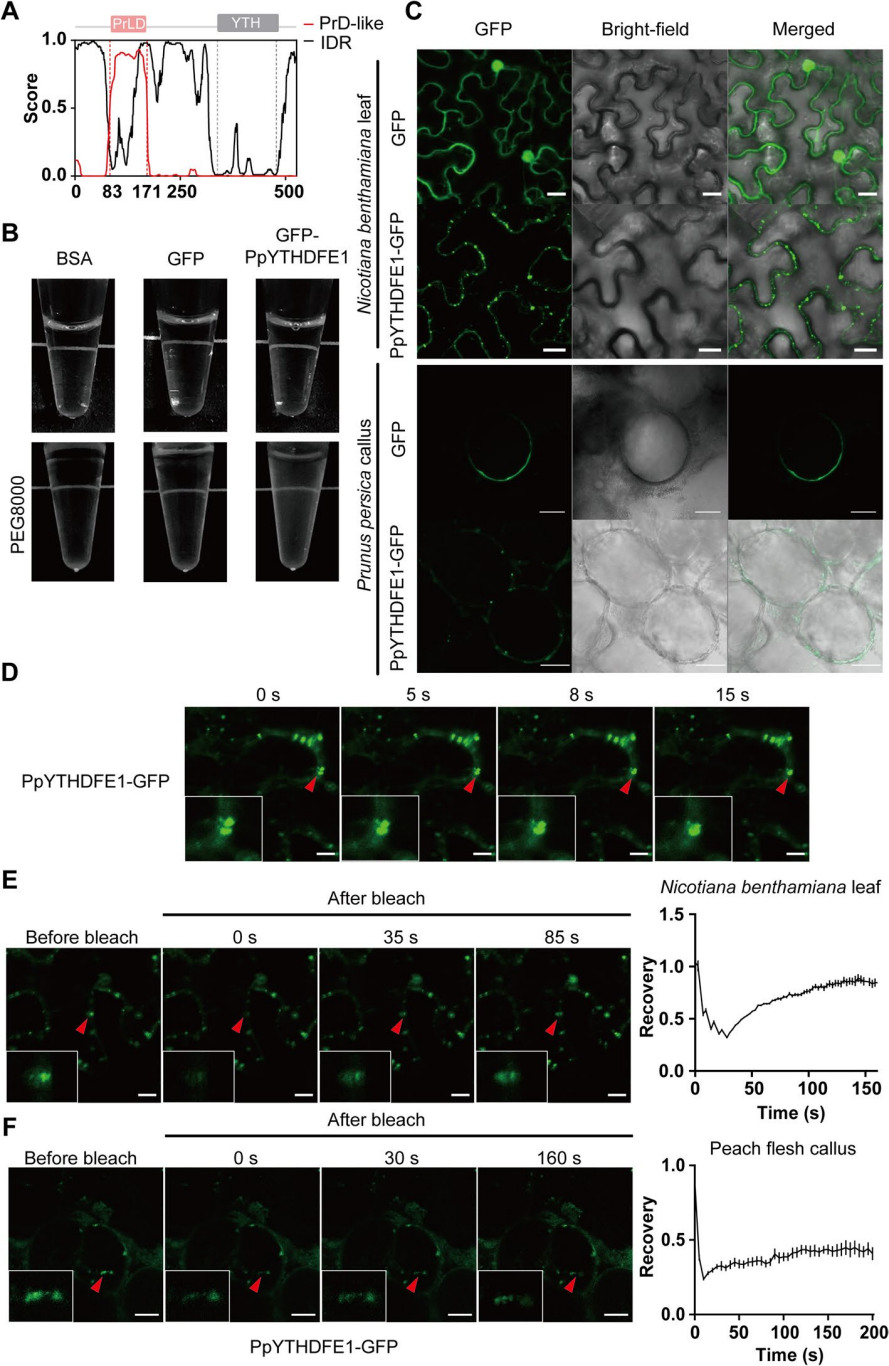
PpYTHDFE1‐GFP can form liquid‐like cytosol condensates. **A** PrLD and IDR domains of PpYTHDFE1 were predicted using “Prion-Like Amino Acid Composition” (PLAAC; http://plaac.wi.mit.edu/) and “Predictor of Natural Disordered Regions” (PONDR; http://pondr.com/), respectively. **B** Visualization of turbid solution of GFP-PpYTHDFE1 proteins under PEG8000 treatment. GFP and bovine serum albumin (BSA) proteins serve as controls. **C** Confocal images of GFP and PpYTHDFE1‐GFP subcellular localization in transgenic peach flesh callus tissue and *Nicotiana benthamiana* leaves (Scale bar, 20 μm). **D** Time-lapse microscopy showing dynamic fusion of PpYTHDFE1‐GFP proteins in transgenic *Nicotiana benthamiana* leaves (Scale bar, 10 μm). The inner box represents the enlarged area pointed to by the red triangle. **E** and **F** shows fluorescence recovery confocal images and recovery curve after photobleaching (FRAP) of PpYTHDFE1‐GFP transgenic *Nicotiana benthamiana* leaves (**E**) and peach flesh callus tissue (**F**) (Scale bar, 10 μm). The inner box represents the enlargement of the FRAP target area pointed to by the red triangle. Time 0 indicates the application time of the photobleaching pulse. The values of recovery curve are mean ± SD (*n* = 3)

Previous studies have established that m^6^A reader proteins orchestrate changes in RNA metabolism through liquid–liquid phase separation (LLPS). To investigate biophysical properties of PpYTHDFE1, we performed structural prediction identifying intrinsically disordered regions (IDRs) and prion-like domain (PrLD, residues 83–171) (Fig. 4A). Using GFP-PpYTHDFE1 recombinant protein, we observed turbidity in sample solutions treated with 20% PEG8000, a phenomenon not seen with GFP protein or BSA (Fig. 4B).

Building on these in vitro observations, we utilized *Agrobacterium*-mediated transient expression systems in *N. benthamiana* and peach flesh callus (Fig. 4C) to investigate localization of PpYTHDFE1 using PpYTHDFE1-GFP. Confocal microscopy revealed PpYTHDFE1-GFP formed dynamic condensates in both systems, contrasting with the diffuse cytoplasmic localization of GFP controls. After deletion of the PrLD domain, PpYTHDFE1△PrLD-GFP loses the ability to form dynamic condensates (Figure S10), suggesting that the PrLD domain plays a crucial role in LLPS. Time-lapse imaging captured rapid fusion events (complete merging within 15 s) demonstrating the fluidity of PpYTHDFE1-GFP condensates (Fig. 4D).

To further investigate the liquid-like characteristics of these cytosolic condensates, fluorescence recovery after photobleaching (FRAP) assays were employed to analyze the dynamics of bleaching events. The results demonstrated fluidity, as shown by the PpYTHDFE1-GFP droplets recovering fluorescence intensity from 30 to 80% within 160 s in tobacco leaves (Fig. 4E). Similar results were observed in peach flesh callus, where the fluorescence intensity of PpYTHDFE1-GFP droplets increased from 25 to 45% after bleaching (Fig. 4F). Collectively, these findings demonstrate that PpYTHDFE1 undergoes LLPS.

### PpYTHDFE1-mediated posttranscriptional silencing of *PpEXP3* governs cold-induced fruit firmness maintenance

**Fig. 5:**
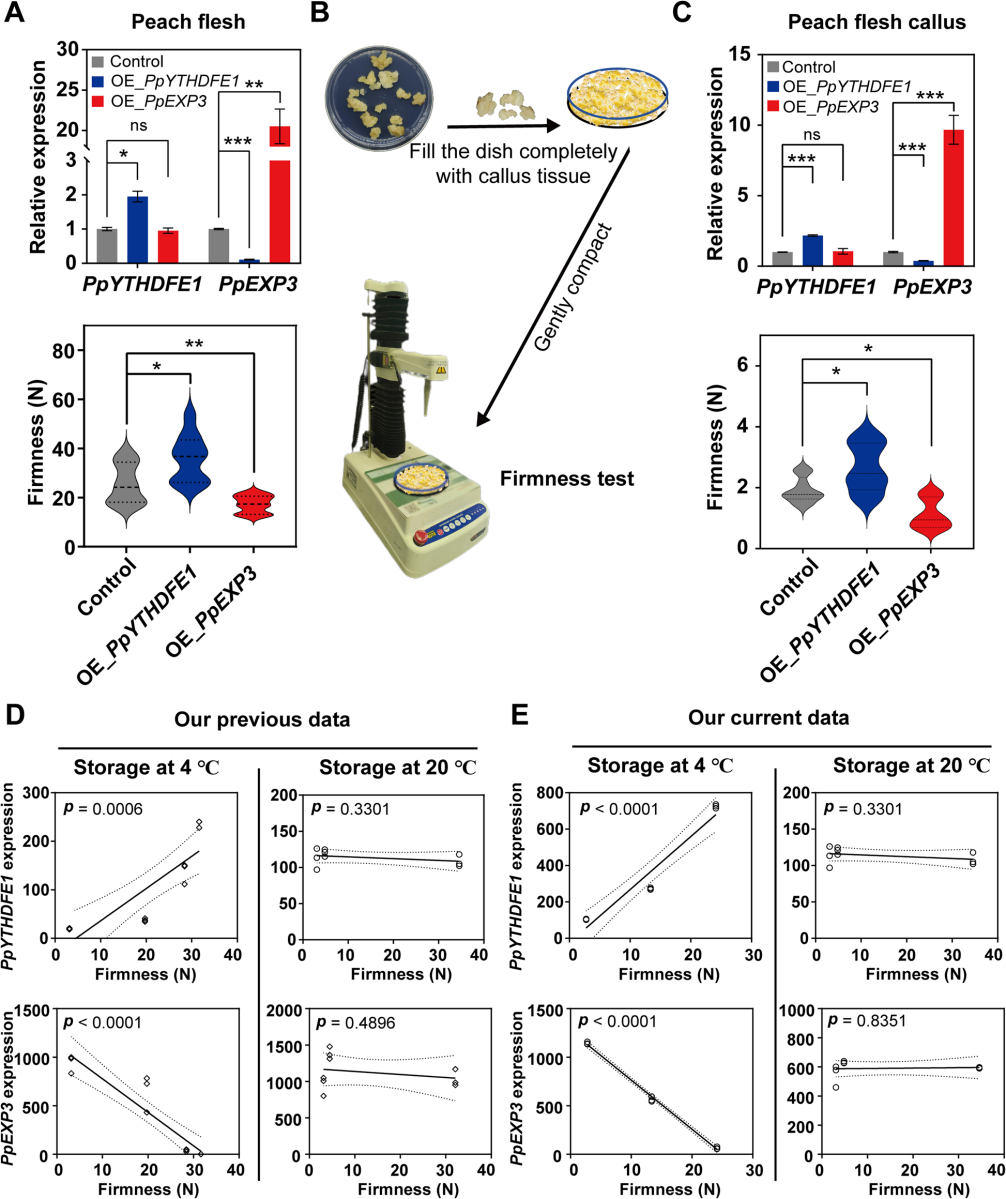
PpYTHDFE1 regulates softening by accelerating mRNA degradation of *PpEXP3* during low temperature. **A** The bar chart displays the expression of *PpYTHDFE1* and *PpEXP3* of peach flesh overexpressing *PpYTHDFE1* and *PpEXP3*. Data are presented as mean ± SD (n = 3 biological replicates). The violin chart displays the firmness results of peach flesh overexpressing *PpYTHDFE1* and *PpEXP3* (*, P < 0.05, **, P < 0.01, ***, *P* < 0.001; Student’s *t* test). ns, no significance. Data are presented as mean ± SD (n = 9 biological replicates). Empty vectors serve as control. **B** Schematic diagram of firmness testing process for peach fruit callus tissue. **C** The bar chart displays the expression of *PpYTHDFE1* and *PpEXP3* of peach flesh callus overexpressing *PpYTHDFE1* and *PpEXP3*. Data are presented as mean ± SD (n = 3 biological replicates). The violin chart displays the firmness results of peach flesh callus overexpressing *PpYTHDFE1* and *PpEXP3* (*, *P* < 0.05, ***, *P* < 0.001; Student’s* t* test). ns, no significance. Data are presented as mean ± SD (n = 9 biological replicates). Empty vectors serve as control. **D** and **E** show correlation between the gene expression of *PpYTHDFE1* and *PpEXP3* with firmness during cold storage, but not during normal storage. Data based on our previous (Duan et al. 2023) (**F**) and current data (**G**). The solid line represents the linear regression line, and the dashed line represents the 95% confidence interval. The *p* value represents the significance of the correlation analysis results. Each point represents an independent sample

Our functional genomics approach revealed PpYTHDFE1 exerts posttranscriptional control over the cell wall-loosening gene *PpEXP3*. To establish phenotypic causality, we performed *Agrobacterium*-mediated transient transformation in peach flesh (cultivar “Hujingmilu”), which demonstrated that twofold *PpYTHDFE1* overexpression suppressed *PpEXP3* transcripts by tenfold (*P* < 0.001) and concomitantly enhanced tissue firmness by 44% (*P* < 0.05) (Fig. 5A). In contrast, *PpEXP3* overexpression reduced firmness by 32% without affecting *PpYTHDFE1* levels, confirming unidirectional regulation. A novel callus-based firmness assay (TA-XT2i plus texture analyzer, 7.9 mm probe) (Fig. 5B) replicated these findings: callus samples overexpressing *PpYTHDFE1* exhibited increased firmness by 39%, while those overexpressing *PpEXP3* displayed a firmness decrease of 41% (Fig. 5C). Notably, no changes in transcript levels were observed for the ethylene synthesis-related gene *PpACO1* after overexpressing *PpYTHDFE1* (Figure S11).

Conservation of this regulatory module was confirmed through development of 35S:*PpYTHDFE1* transgenic tomatoes (T2 generation) showing 45% higher fruit firmness (*P* < 0.001) (Figure S12A), without affecting ethylene production (Figure S12B). “Hujingmilu” is a melting peach cultivar, and we want to discover if the low temperature regulation of *PpYTHDFE1* and *PpEXP3* is conserved in non-melting peach cultivars, so we determined their expression patterns in fruit of the non-melting peach cultivar “Qingzhoumi” during low-temperature storage. The results showed that low temperature could significantly maintain fruit firmness (Figure S13A), while activating *PpYTHDFE1* expression and inhibiting *PpEXP3* transcription levels (Figure S13B) revealing the conservation of this low temperature response mechanism. Meta-analysis revealed cold-specific correlation patterns: *PpYTHDFE1* expression positively correlated with firmness (*P* < 0.001) while *PpEXP3* showed negative correlation (*P* < 0.001) exclusively under cold storage (Fig. 5D, E).

## Discussion

**Fig. 6:**
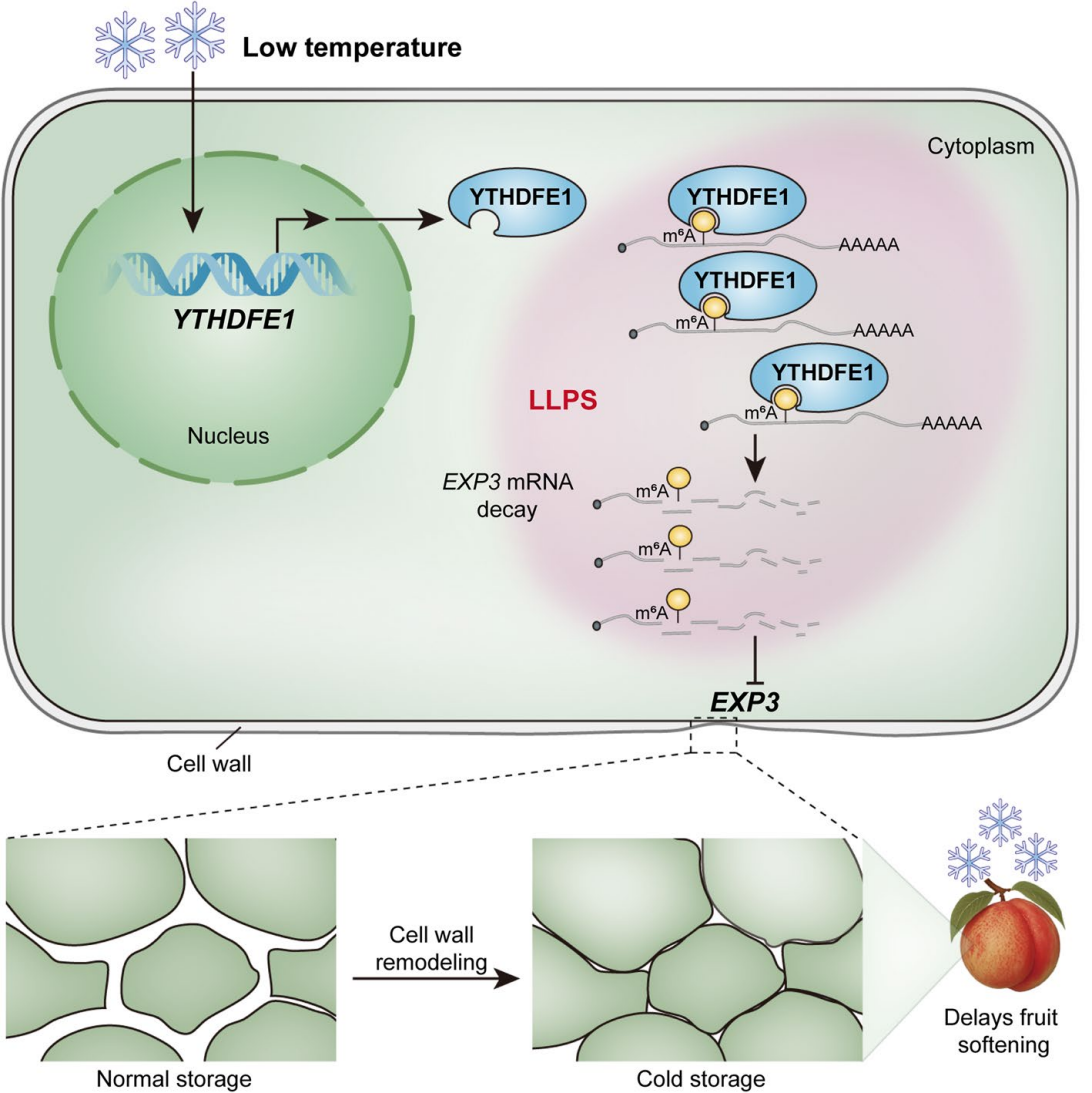
Model for the m^6^A reader PpYTHDFE1 in regulation of peach fruit firmness. During low-temperature storage, the expression of PpYTHDFE1 increases and undergoes phase separation. PpYTHDFE1 selectively binds to m^6^A-modified *PpEXP3* transcripts, a cell wall-loosening gene associated with fruit firmness, and accelerates the degradation of their transcripts. This interaction reduces the abundance of *PpEXP3* transcripts, causing cell wall remodeling, thereby maintaining peach fruit firmness

Low temperature exerts profound effects on plant adaptation, growth, and postharvest physiology, particularly in perishable commodities such as fruits. While refrigeration delays softening by suppressing cell wall disassembly, the molecular mechanisms underlying this process are not fully understood. In peach, Wang et al. showed that low temperature caused major reductions in transcripts encoding enzymes involved in cell wall modification and ethylene biosynthesis and that this was correlated with firmness (Wang et al. 2017c). The mechanism of this reduction in transcripts was not studied, although there is evidence that often changes in transcript levels are modulated by transcription factors (Shi et al. 2022). Our integrative transcriptomic and epitranscriptomic reveals a novel regulatory axis in which the YTHDF-type m^6^A reader PpYTHDFE1 couples low-temperature sensing with posttranscriptional control through LLPS, selectively destabilizing transcripts encoding cell wall-loosening proteins such as *PpEXP3* (Fig. 6).

Central to this regulatory paradigm is the identification of *PpEXP3*, an expansin gene whose transcript undergoes m^6^A modification and temperature-dependent decay during postharvest storage (Figs. 3 and 5). Phylogenetic and coexpression analyses across species highlight the conserved role of EXP family proteins as nonenzymatic facilitators of cell wall loosening (Cosgrove 2024). For instance, coexpression network analysis across 604 peach RNA-seq datasets indicates the role that *PpEXP3* plays in softening dynamics (De Los Cobos et al. 2024), and contributes to the breakdown of the cell wall matrix (Shi et al. 2023). Apple fruit firmness is genetically controlled by *MdEXP-A1* (Su et al. 2025), while *SlExp1* and *SlCel2* synergism drive tomato softening (Su et al. 2024), although pectin metabolism also plays a role in softening (Shi et al. 2023; Ortega‐Salazar et al*.*, 2024). Interestingly, *EXPs* expression demonstrates remarkable environmental plasticity through organ-specific regulation. For example, downregulation in sweet potato leaves and roots (Noh et al. 2009) versus upregulation in Arabidopsis seeds (Yamauchi et al. 2004) and rice anthers (Imin et al. 2004). Such flexibility enables precise cell wall remodeling under different developmental and stress conditions, including salinity responses mediated by *EXP* transcriptional reprogramming (Kwon et al. 2008; Zhang et al. 2014). It is also evolutionarily conserved in Arabidopsis, where the ortholog AtECT8 destabilizes *AtEXPA6* under salinity (Cai et al. 2024). This cross-species parallelism positions m^6^A readers as a universal environmental sensor fine-tuning cell wall dynamics under abiotic stress. Here, we demonstrate that cold-induced *PpEXP3* degradation is mediated by the m^6^A reader PpYTHDFE1, and this mechanism is conserved between cultivars (Figure S13). Moreover, only PpYTHDFE1 responsed to cold while seven other readers were unaffected by cold (Fig. 1D).

Previous work has shown that the CBF-dependent cold response in plants involves transcriptional regulation of COR genes (Xie et al. 2018; Mei et al. 2023). In peach, we observed a dual-layered regulatory mechanism: transcriptional activation of COR genes coupled with epitranscriptomic fine-tuning transcript levels. Specifically, 46.7% (49/105) of CBF targets are marked by m^6^A modifications (Table S7). *PpCBF6* demonstrates thermosensitive induction kinetics, achieving maximal activation after seven days storage at 4 ℃, followed by a return to basal levels upon exposure to ambient temperature (Fig. 2B). Further investigation is warranted to determine whether m^6^A reader influences the stability of COR gene transcripts, and to elucidate the mechanism by which low temperature induces the expression of m^6^A reader *PpYTHDFE1*. Intriguingly, PpYTHDFE1 plays a broader role beyond cell wall remodeling, as evidenced by the enrichment of m^6^A transcripts in processes such as ubiquitination, kinase signaling, and stress hormone responses (Figure S5; Tables S5, S6), suggesting its possible role as a multifunctional hub in other genetic cold adaptation responses.

During peach fruit ripening at ambient temperature, ethylene biosynthesis genes (e.g., *PpACS1* and *PpACO1*) are transcriptionally activated by PpNAC1, driving climacteric ethylene production, subsequent cell wall disassembly and other ripening-related events (Cao et al. 2024a). However, neither endogenous ethylene surges, exogenous ethylene treatment, or 1-MCP altered *PpYTHDFE1* expression (Wang et al. 2025) (Figure S7), demonstrating its decoupling from canonical ethylene signaling cascades. Strikingly, cold storage suppressed ethylene production by inhibiting the expression of *PpACS1* and *PpACO1* and this has been noted previously in peach (Duan et al. 2023; Wang et al. 2017c). Importantly, the PpYTHDFE1-mediated mRNA decay described here, specifically targeting the cell wall-loosening gene *PpEXP3* via m^6^A recognition, occurred independent of ethylene (Fig. 2F). Peach fruit flesh and callus overexpressing *PpYTHDFE1* led to 44% and 39% increase in firmness (Fig. 5) but did not change the expression of the key ethylene synthesis gene *PpACO1* (Figure S11), which is consistent with the result that PpYTHDFE1 does not recognize *PpACO1* (Fig. 2F). Similarly, transgenic tomatoes overexpressing *PpYTHDFE1* exhibited a 45% increase in fruit firmness, without perturbing ethylene production or the ripening progression (Wang et al. 2025) (Figure S12). These findings reveal a previously unrecognized regulatory axis in which epitranscriptomic control of cell wall loosening gene such as *PpEXP3* via a specific cold-induced m^6^A reader operates parallel to hormonal pathways. While ethylene-mediated transcriptional regulation of fruit ripening and softening has been extensively characterized (Seymour et al. 2013; Shi et al. 2023; Li et al. 2020), our study suggests an ethylene-independent pathway for postharvest textural regulation, and provides a translatable framework for engineering shelf-life extension in fruits without compromising ripening progress. However, the effect of cold on other aspects of fruit quality need to be considered (Duan et al. 2023).

Our findings redefine the paradigm of postharvest biology by integrating epitranscriptomics with environmental sensing. The discovery that m^6^A readers act as thermosensitive regulators of cell wall dynamics opens avenues for engineering climate-resilient crops and reducing postharvest losses. For instance, targeting YTH-EXP nodes could decouple shelf-life extension from undesirable traits such as delayed ripening, representing a critical advance for climacteric fruits. By elucidating how low temperature reshapes the epitranscriptome, this study bridges molecular insight with translational potential, thereby advancing sustainable horticulture in an era of climate uncertainty. Moreover, the evolutionary conservation of this mechanism suggests its potential broad applicability across various other abiotic stresses, including salinity and drought.

## Methods

### Plant materials and treatments

White-fleshed melting flesh peach fruits (*Prunus persica* L. Batsch., cv. Hujingmilu) harvested at commercial maturity from an orchard in Ningbo, Zhejiang Province, China, were used in this study. Uniform fruits without visual defects were selected for postharvest storage. The peaches were randomly divided into two groups and stored at 4 ℃ with a relative humidity of 90% for 0 and 7 days, labeled as C0d and C7d, respectively. Randomly selected halves from each group were then transferred to a 20 ℃ environment for an additional 3 days of storage, labeled as C0dS3 and C7dS3. White-fleshed non-melting flesh peach fruits (*Prunus persica* L. Batsch., cv. Qingzhoumi) harvested at commercial maturity were obtained from an orchard in Linyi, Shandong Province, China. Uniform fruits without visual defects were selected for postharvest storage. The peaches were randomly divided into two groups and stored at 4 ℃ and 20 ℃ with a relative humidity of 90% for 5 days, respectively. Flesh tissue slices, approximately 5 mm thick, were collected at each time point, frozen in liquid nitrogen, and stored at −80 ℃ for biochemical and molecular analysis. Three biological replicates were prepared, with at least five fruits used per replicate.

### Fruit ethylene production and firmness analysis

Ethylene production was measured as described previously (Duan et al. 2023). The fruits were enclosed in a 1.8 L airtight container for one hour to analyze ethylene production. A 1 mL gas sample from the container was then injected into a gas chromatograph (GC) equipped with a flame ionization detector (Agilent Technologies 6890 N GC System, CA, USA). The temperature for the oven, injector, and detector were set to 100, 140, and 230 ℃, respectively. Fruit firmness was assessed using a texture analyzer (TA-XT2i plus, Stable Micro System, UK) with a 7.9 mm diameter probe. The puncture depth was 10 mm, and the puncture speed was 1 mm/s. Each fruit was measured twice, with a total of no less than 9 fruits measured. For transient overexpressing peach fruit flesh and callus, the puncture depth was 5 mm, and the puncture speed was 1 mm/s. Each tissue was measured twice, with a total of no less than 9 pieces of tissue measured.

### Phylogenetic analysis and structural characterization

The phylogenetic tree was constructed using MEGA X software (Kumar et al*.*, 2018), with parameters set to the Neighbor-Joining (NJ) method and Poisson correction, along with pairwise deletion and 1000 bootstrap repetitions. Multiple sequence alignment of all YTH-domain proteins was performed using Clustal X 2.0. The AI-predicted secondary structure of proteins was downloaded from the AlphaFold Protein Structure Database (https://alphafold.ebi.ac.uk/) (Tunyasuvunakool et al. 2021).

### Protein expression and RNA electrophoretic mobility shift assay (RNA-EMSA)

The coding sequences of *PpYTHDFE1* were cloned from the cDNA of Hujingmilu and inserted into the pGEX-*GST* vector. The recombinant vectors were then introduced into *Escherichia coli* strain Rosetta (DE3). A GST-tag Protein Purification Kit (Beyotime, China) was used to purify the proteins according to the manufacturer's instructions. All primers used for vector construction are listed in Table S11.

To assay the binding affinity of GST-PpYTHDFE1 and GST-PpYTHDFE1m to m^6^A modifications, labeled RNA probes were designed with m^6^A modifications; detailed sequence information of the probes is provided in the Table S12. The RNA EMSA was conducted using the LightShift Chemiluminescent RNA EMSA Kit (Thermo Fisher Scientific, USA), following the manufacturer's instructions. In the RNA EMSA systems, the labeled probes were added at a concentration of 10 nM, with a gradient of decreasing protein content.

### m^6^A-seq and data analysis

The m^6^A-seq was performed as previously described (Bian et al. 2024). Briefly, total RNAs were extracted from the pericarp tissues of peach fruit at the C0d, C7d, C0dS3, and C7dS3 stages described above. RNA integrity was assessed using an Agilent 2100 bioanalyzer (Agilent, G2939A). mRNAs were then purified using a DynabeadsTM mRNA purification kit (Invitrogen, USA) and fragmented into approximately 200-nucleotide-long pieces by incubating for 10 min in RNA fragmentation buffer (10 mM Tris–HCl, 10 mM ZnCl_2_, pH 7.0). The fragmentation reaction was halted by adding 100 mM EDTA, and the fragments were recovered by isopropanol precipitation. A total of 500 ng of fragmented mRNAs were preserved as the input control.

For m^6^A immunoprecipitation (m^6^A-IP), 5 μg of fragmented mRNAs were incubated with 2 μg of anti-m^6^A polyclonal antibody (Synaptic Systems, Germany) in 400 μL of immunoprecipitation (IP) buffer containing 10 mM Tris-HCI, pH 7.4, 150 mM NaCl, 0.1% IGEPAL CA-630 (v/v), and 300 U/mL RNase inhibitor (Takara, Japan). After mixing at 4 ℃ for 2 h, Dynabeads Protein-A (Thermo Fisher Scientific, USA) were washed and added for immunoprecipitation, followed by elution with 6.7 mM N^6^-methyladenosine (Sigma, USA) in the IP buffer to release the bound mRNAs from the beads. The eluted mRNAs were recovered by isopropanol precipitation (50% isopropanol (v/v) and 136 mM CH_3_COONa, PH 5.2) overnight at 4 ℃. A total of 50 ng of immunoprecipitated mRNAs or input control was used for library construction using the NEBNext Ultra II RNA Library Prep Kit (NEB, USA). High-throughput sequencing was conducted on the Illumina NovaSeq 6000 with a paired-end read length of 150 bp following standard protocols. The m^6^A-seq analysis was performed with three independent biological replicates, each comprising a mixture of at least five peach fruits.

The quality of raw reads from m^6^A-seq was assessed using the FastQC tool. Reads showing adapter contamination, low-quality bases, or fewer than 30 nucleotides in length were removed using Trimmomatic (Bolger et al. 2014). HISAT2 (Kim et al. 2015) was then clean reads were aligned to the to the reference genome of *Prunus persica* v2.1. Mapped reads from the IP and input libraries were used for peak calling with the R package exomePeak2 (Meng et al. 2014). The identified peaks were annotated using the same package. We identified m^6^A peaks with an estimated false discovery rate (FDR) of less than 0.05. Only m^6^A peaks consistently detected across all three biological replicates for each sample were designated as high-confidence m^6^A peaks for further analysis. Conserved sequence motifs of m^6^A peaks were identified using HOMER software (http://homer.ucsd.edu/homer/), and the m^6^A peaks were visualized using IGV software (http://www.igv.org/).

### RIP-qPCR

The RIP-qPCR was performed as previously described (Bian et al. 2024). Fruit samples at the C7d stage were fixed using a 1% formaldehyde solution, followed by termination of fixation with 0.125 M glycine solution. The fixed tissues were ground into powder, and 5 g of this powder was dissolved in 7.5 mL of lysis buffer (50 mM Tris–HCl, pH 7.5, 300 mM KCl, 5 mM MgCl_2_, 5 mM DTT, 0.2% Triton X-100, 2% glycerol, 1 mM PMSF, 1 × Protease Inhibitor, 160 U/mL RNase inhibitor). The samples were lysed at 4 ℃ for 30 min, then centrifuged to collect the supernatant. Ten percent of the total supernatant was used as input. The remaining supernatant was divided into two equal parts, to which equal amounts of polyclonal anti-PpYTHDFE1 antibody (Abmart, China) or IgG (ABclonal, China), conjugated to Pierce™ Protein A magnetic beads (Thermo Fisher Scientific, USA), were added and incubated overnight at 4 ℃ for immunoprecipitation. The beads were washed five times using washing buffer (25 mM Tris–HCl, pH 7.5, 150 mM NaCl, 0.05% Tween-20, 1 mM PMSF, 1 × Protease Inhibitor, 80 U/mL RNase inhibitor). After digestion with proteinase K and DNase I, RNA was extracted using RNAiso Plus (Takara, Japan). The extracted RNA served as a template for complementary DNA (cDNA) synthesis using HiScript II Q RT SuperMix for qPCR (+ gDNA wiper) (Vazyme, #R223). Quantitative real-time PCR (qRT-PCR) was performed with ChamQ Universal SYBR qPCR Master Mix (Vazyme, Q711) on the CFX96 Real-Time PCR System (Bio-Rad, Hercules, CA, USA). This assay was conducted with three independent biological replicates. All primers used for RIP-qPCR are listed in Table S11.

### Transient overexpression in callus mediated by *Agrobacterium tumefaciens*

The method for gene transient overexpression in callus was adapted from Ni et al. with modifications (Ni et al. 2023). Primers specified in Table S11 were used to clone the complete coding sequences (CDS) of target genes into the pCambia1300-221 vector. These recombinant vectors, along with an empty vector control, were transformed into *Agrobacterium tumefaciens* GV3101 via chemical transformation. Peach callus was induced from the flesh tissue of *Prunus persica* L. Batsch cv. Hujingmilu mature fruits. The callus was carefully transferred to a bacterial suspension (OD600 = 0.8) and soaked for 10 min. The mixture was then filtered through three layers of cheesecloth, and any residual bacterial suspension on the callus surface was absorbed using sterile filter paper. The infected callus was transferred to solid SH medium containing 20 mg/L AS, with filter paper laid on the surface. After 3 days of co-culture incubation in the dark at 24 °C and 60% relative humidity, the infected callus was transferred to solid SH medium containing 50 mg/L kanamycin (Kan) for screening. Cultures were maintained in the dark for 4 days before sampling. The solid SH medium composition was: 13.2 g/L SH, 20 g/L sucrose, 1 g/L PVP, and 7 g/L agar.

### Transient RNAi silencing in callus mediated by *Agrobacterium tumefaciens*

A 400 bp fragment from the coding region of *PpYTHDFE1* was cloned using appropriate primers (Table S11) and inserted into the pHELLSGATE2 gene silencing vector via Gateway technology. The sequence-verified recombinant vector, along with the empty vector, was transferred into *Agrobacterium tumefaciens* strain GV3101. The callus was infected as described in the transient overexpression section above. After one week of infection, the callus was frozen in liquid nitrogen and stored at −80 ℃ for biochemical and molecular analysis.

### Transient overexpression in fruit flesh mediated by *Agrobacterium tumefaciens*

Transient overexpression in peach fruit was performed according to our previous study (Cao et al. 2024b). Primers specified in Table S11 were used to clone the complete coding sequence (CDS) of target genes into the pCambia1300-221 vector. The recombinant vectors and an empty vector control were transformed into *Agrobacterium tumefaciens* GV3101 via chemical transformation and cultured at 28 ℃ until the OD600 reached 0.8. After disinfecting the surface of the peach fruit with 0.5% NaClO for 10 min, two flesh cubes (0.8 cm thick) were taken from opposite sides of each fruit. *A. tumefaciens* carrying the target genes or the empty vector was infiltrated into the flesh under a vacuum of 70 kPa. Then the tissue was rinsed three times with sterile water and cultured on Murashige and Skoog (MS) medium for 3 days in a growth chamber (25 ℃, 85% RH). The flesh cubes were then sampled for firmness analysis. Transient expression treatments were repeated three times with three fruits each time.

### RNA isolation and quantitative real-time PCR (qRT-PCR) analysis

Total RNA was extracted from samples using the FastPure Universal Plant Total RNA Isolation Kit (Vazyme, China). The extracted RNA was then used to synthesize cDNA with the HiScript II Q Select RT SuperMix for qPCR (+ gDNA wiper) (Vazyme, China). Quantitative RT-PCR was performed on the CFX96 system (Bio-Rad, Hercules, California, USA) using ChamQ SYBR qPCR Master Mix (Vazyme, China). *PpTEF2* (Prupe.4G138700) was used as the internal control. Gene expression levels were quantified using the 2^−ΔΔCt^ method as described by(Schmittgen & Livak 2008). A list of primers is provided in Table S11.

### mRNA stability assay

The mRNA stability assay was performed as previously described (Bian et al. 2024), using *Nicotiana benthamiana* leaf and callus derived from peach fruit flesh transiently overexpressing *PpYTHDFE1*, with an empty vector used as a control. The leaves and fruit callus were infiltrated with a solution containing 20 μg/mL actinomycin D. After vacuum infiltration for 1 min and incubation for 5 min, samples were collected as time 0 controls, and additional samples were collected at 1, 2, and 4 h. The mRNA levels of the genes were then examined by qRT-PCR, as described above. All primers used for PCR amplification are listed in the Table S11. The data analysis adopts the nonlinear regression one phase decay method by GraphPad Prism 9.5 software.

### LLPS assay

The LLPS assay method was based on that of Luo et al. (Luo et al. 2023), with some modifications. To express and purify GFP-PpYTHDFE1 recombinant proteins, the CDS of *PpYTHDFE1* was cloned and inserted into the pET-N expression vector containing N-terminal GFP. Primers used for vector construction are listed in Table S11. The vector was transformed into *E. coli* Rosetta (DE3), and GFP-PpYTHDFE1 protein expression was induced by adding 0.1 mM isopropyl β-d-thiogalactopyranoside (IPTG) at 16 °C for 20 h. Cells were harvested and lysed by sonication in a solution containing 25 mmol/L HEPES (pH 7.4) and 200 mmol/L NaCl. After centrifugation at 10,000 g for 40 min at 4 °C, the supernatants were passed through a HisTALON Gravity Column (Takara, Japan). Bound proteins were eluted with elution buffer (25 mmol/L HEPES (pH 7.4), 200 mmol/L NaCl, and 150 mmol/L imidazole). To observe the in vitro phase separation ability of GFP-PpYTHDFE1, the purified proteins were diluted to 10 μM and mixed with 20% (m/v) PEG 8000 at a 1:1 ratio, using BSA protein and GFP protein as controls.

To observe the phase separation ability of PpYTHDFE1 in vivo, transiently overexpressed tobacco leaves and callus were observed under a Zeiss LSM880 confocal laser scanning microscope (Carl Zeiss AG, Germany) equipped with × 20, × 40, and × 60/oil objectives. GFP fluorescence was excited at 488 nm and detected at 490–535 nm.

For time-lapse microscopy, GFP fluorescence was observed under the same microscope with images captured every second for 5 min and analyzed using ZEISS ZEN lite software. For the FRAP assay, GFP fluorescence was bleached using a 488 nm laser pulse at 70% intensity. Fluorescence recovery was recorded every second for 5 min.

### Statistical analysis

Significance analyses were conducted using GraphPad Prism 9.5 software. Pairwise comparisons were performed using an unpaired *t*-test. Multiple comparisons were analyzed through ANOVA followed by Tukey’s test, with statistically significant differences (*P* < 0.05) indicated by different lowercase letters. The fitting model employed for RNA stability analysis is based on the one-phase decay model provided by GraphPad Prism 9.5 software. Correlation analyses were conducted using GraphPad Prism 9.5 software, calculating Pearson correlation coefficients.

## Supplementary Information

Additional file 1: Figure S1. PpYTHDFE1 is a low-temperature induced m^6^A reader. Figure S2. Structure for m^6^A binding of AtECT8 and PpYTHDFE1 simulated by AlphaFold2. Figure S3. Characteristics of m^6^A localization and sequence motif in peach fruit. Figure S4. Presence of m^6^A stabilizes gene expression during peach storage. Figure S5. Venn diagram showing the overlap genes of DEGs increased from C0d to C7d, DEGs decreased from C7d to C7dS3 and m6A genes. Figure S6. m^6^A methylation status of *PpACO1* during storage were shown by Integrative Genomics Viewer (IGV). Figure S7. Expression of *PpYTHDFE1* in peach fruit under ethylene and 1-MCP treatment for 72h. Figure S8. Correlation analysis of the expression of *PpYTHDFE1* and *PpEXP3* during cold storage. Figure S9. Expression of *PpYTHDFE1* of peach flesh callus after storage at 4 ℃ or 20 ℃ for 3 and 7 days. Figure S10. PpYTHDFE1∆PrLD‐GFP can not form liquid‐like cytosol condensates. Figure S11. Expression of *PpACO1* in peach flesh callus overexpressing *PpYTHDFE1*. Figure S12. Overexpressing *PpYTHDFE1* increases fruit firmness but not ethylene production. Figure S13. Low temperature activation of *PpYTHDFE1* expression and inhibition of PpEXP3 expression are also conserved in non-melting peach.

Additional file 2: Table S1. Basic information of m^6^A-seq in this study. Table S2. m^6^A density in actively expressed transcripts. Table S3. GO enrichment results of 2553 intersection DEGs in figure 2A. Table S4. Expression profiles of 2553 intersection DEGs in Figure 2A. Table S5. Expression profiles of 2198 intersection DEGs in Figure S5. Table S6. GO enrichment results of 2198 intersection DEGs in figure S5. Table S7. Expression patterns and m^6^A modification status of CBF genes and their downstream COR genes in peach. Table S8. Expression patterns and m^6^A modification profiles of peach transcription factors. Table S9. Expression patterns and m^6^A modification profiles of peach hormonal mediators. Table S10. Homologous cell wall-related genes that have been characterized in various plants and are known to affect fruit firmness. Table S11. Primer sequences used in research. Table S12. Probe sequences used in research.

## Data Availability

The datasets supporting the conclusions of this article are available in the Genome Sequence Archive (Genomics, Proteomics & Bioinformatics 2021) in National Genomics Data Center (Nucleic Acids Res 2022), China National Center for Bioinformation/Beijing Institute of Genomics, Chinese Academy of Sciences (GSA: CRA024139) that are publicly accessible at https://ngdc.cncb.ac.cn/gsa.

## Abbreviations

1-MCP: 1-Methylcyclopropene
CBF: C-repeat Binding Factor
CEL: Cellulases
COR: Cold-Regulated Genes
DEGs: Differentially expressed genes
ECT: Evolutionarily conserved C-terminal regions
EMSA: Electrophoretic mobility shift assays
endo-PGs: Endo-Polygalacturonases
EXP: Expansin
FLW: Food loss and waste
FRAP: Fluorescence recovery after photobleaching
GO: Gene Ontology
IDRs: Intrinsically disordered regions
LLPS: Liquid-liquid phase separation
m^6^A: N^6^-methyladenosine
PG: Polygalacturonase
PL: Pectate lyase
PrLD: Prion-like domain
TFs: Transcription factors
YTH: YT512-B homology
