# Supplementary material for: Cold storage delays peach fruit softening via m6A reader PpYTHDFE1 liquid-liquid phase separation-mediated degradation of cell wall-loosening transcript PpEXP3
Source: Mol Hortic. 2026 May 9;6:33. doi: 10.1186/s43897-025-00218-3 (PMC13156859; doi:10.1186/s43897-025-00218-3)
Supplement: Supplementary file 1 — Additional file 1: Figure S1. PpYTHDFE1 is a low-temperature induced m6A reader. Figure S2. Structure for m6A binding of AtECT8 and PpYTHDFE1 simulated by AlphaFold2. Figure S3. Characteristics of m6A localization and sequence motif in peach fruit. Figure S4. Presence of m6A stabilizes gene expression during peach storage. Figure S5. Venn diagram showing the overlap genes of DEGs increased from C0d to C7d, DEGs decreased from C7d to C7dS3 and m6A genes. Figure S6. m6A methylation status of PpACO1 during storage were shown by Integrative Genomics Viewer (IGV). Figure S7. Expression of PpYTHDFE1 in peach fruit under ethylene and 1-MCP treatment for 72h. Figure S8. Correlation analysis of the expression of PpYTHDFE1 and PpEXP3 during cold storage. Figure S9. Expression of PpYTHDFE1 of peach flesh callus after storage at 4 ℃ or 20 ℃ for 3 and 7 days. Figure S10. PpYTHDFE1∆PrLD‐GFP can not form liquid‐like cytosol condensates. Figure S11. Expression of PpACO1 in peach flesh callus overexpressing PpYTHDFE1. Figure S12. Overexpressing PpYTHDFE1 increases fruit firmness but not ethylene production. Figure S13. Low temperature activation of PpYTHDFE1 expression and inhibition of PpEXP3 expression are also conserved in non-melting peach. [file 43897_2025_218_MOESM1_ESM.docx]

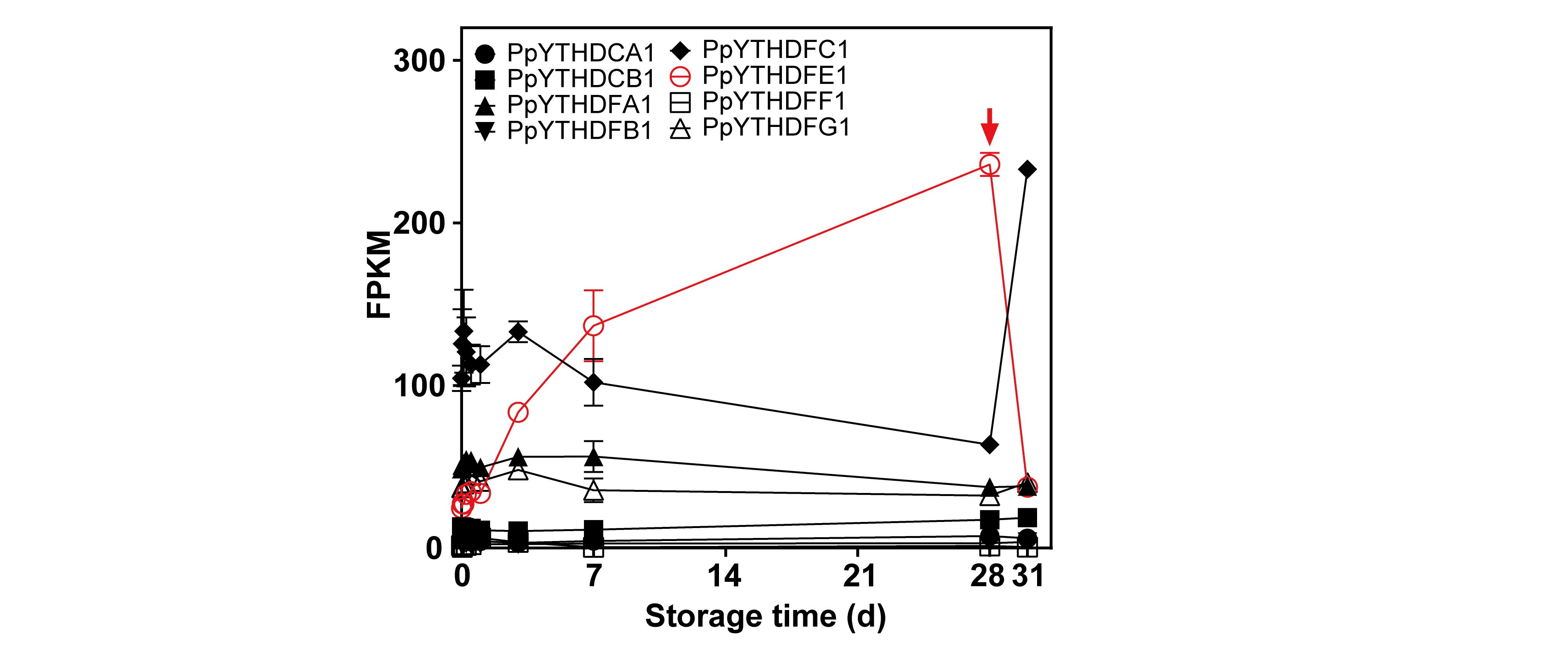


**Fig. S1** **PpYTHDFE1 is a low-temperature induced m^6^A reader.** Data are presented as mean ± SD of three independent biological replicates. FPKM, Fragments Per Kilobase per Million. The red arrow represents the transfer from low-temperature storage to a 20 ℃ shelf.


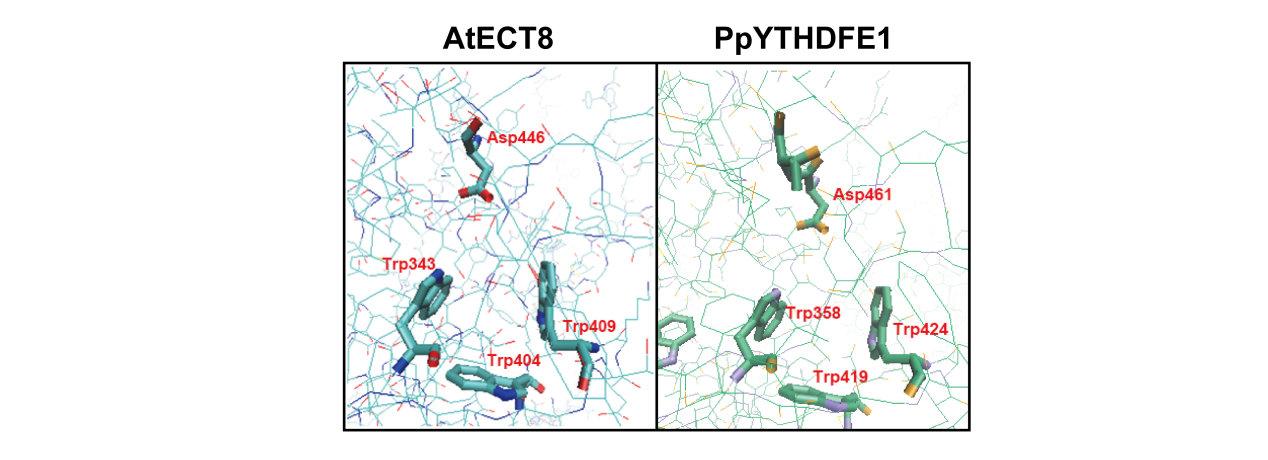


**Fig. S2** **Structure for m^6^A binding of AtECT8 and PpYTHDFE1 simulated by AlphaFold2.** A conservative aromatic cage structure is dominated by tryptophan.


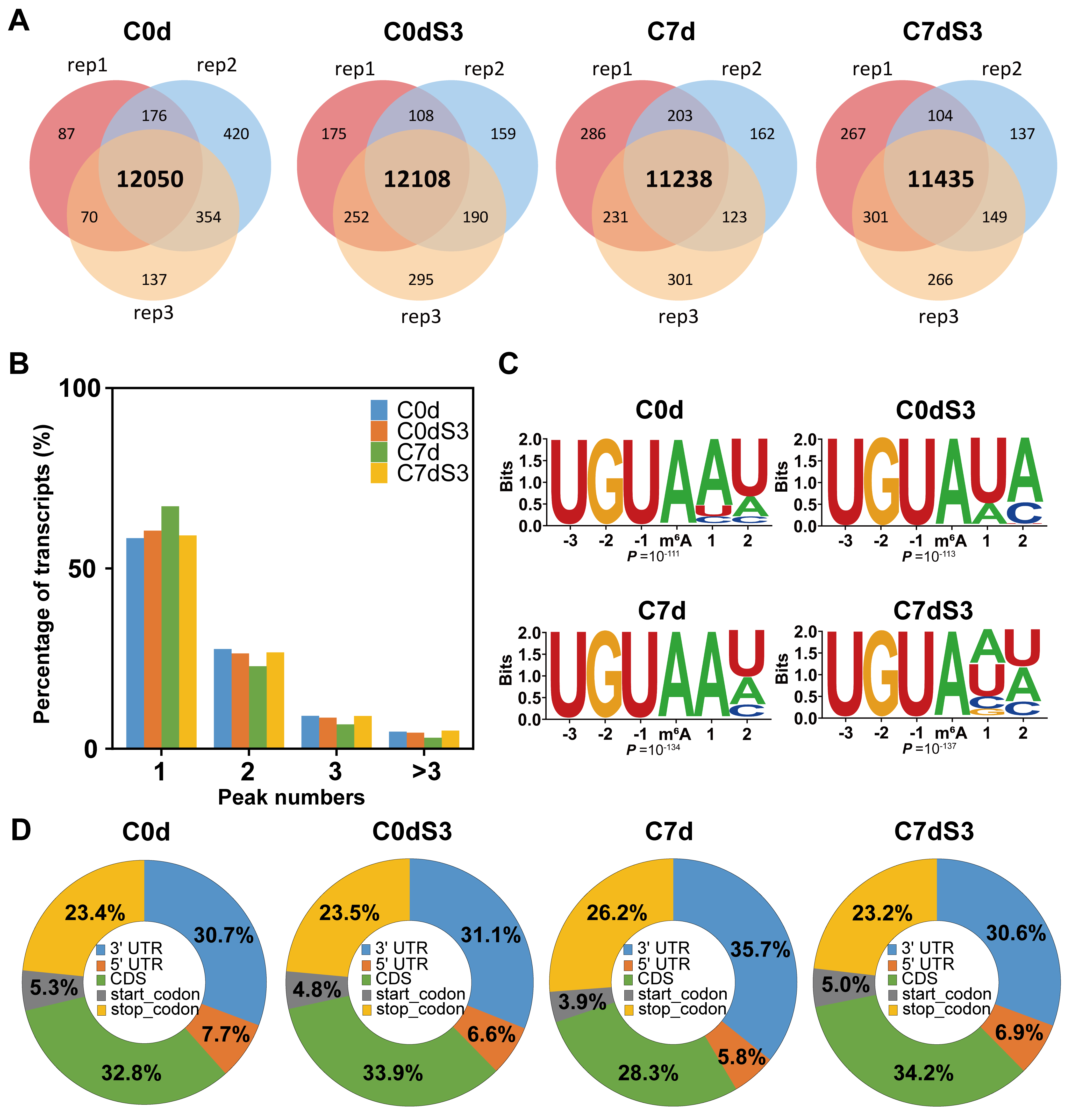


**Fig. S3** **Characteristics of m^6^A localization and sequence motif in peach fruit.** (A) Venn diagrams depicting the overlap of m^6^A peaks from three independent m^6^A-seq experiments on fruit at the four storage stages. rep, replicate. (B) Percentage of the m^6^A-containing transcripts containing various m^6^A peak numbers among samples. (C) Sequence motif identified within m^6^A peaks by HOMER software of different storage stages. The p value represents the significance of the correlation analysis results. (D) Distribution of m^6^A peaks in five non-overlapping transcript segments. UTR, untranslated region; CDS, coding sequence. C0d, peach fruit at harvest; C0dS3, fruit stored at 20 ℃ for 3 days; C7d, fruit following 7 days of cold storage; C7dS3, fruit stored in the cold for 7 days followed by a 3 days recovery at 20 ℃.


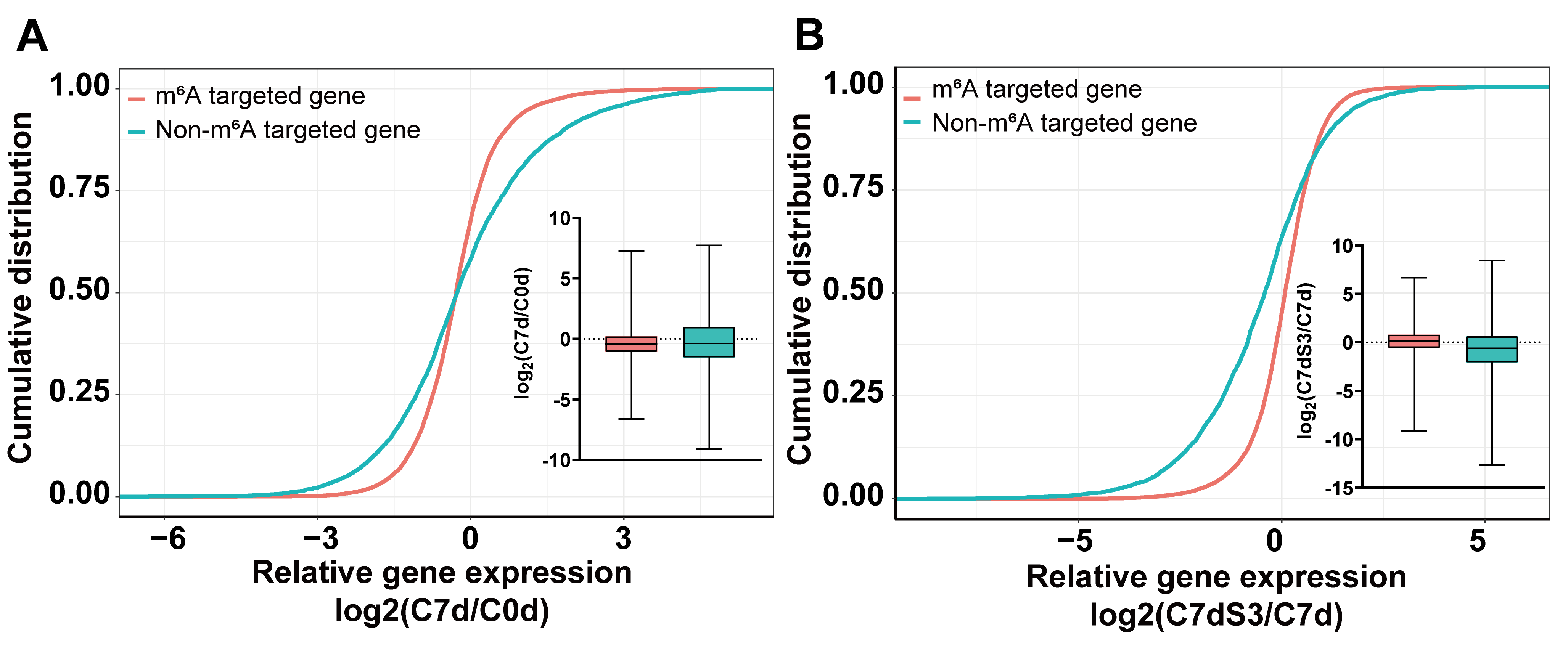


**Fig. S4** **Presence of m^6^A stabilizes gene expression during peach storage.** (A) and (B) show cumulative distribution and boxplot of relative mRNA abundance of m^6^A targeted gene and non-m^6^A targeted gene during C0d to C7d (A), and C7d to C7dS3 (B), respectively. Data in boxplot are presented as mean ± SD of three independent biological replicates.


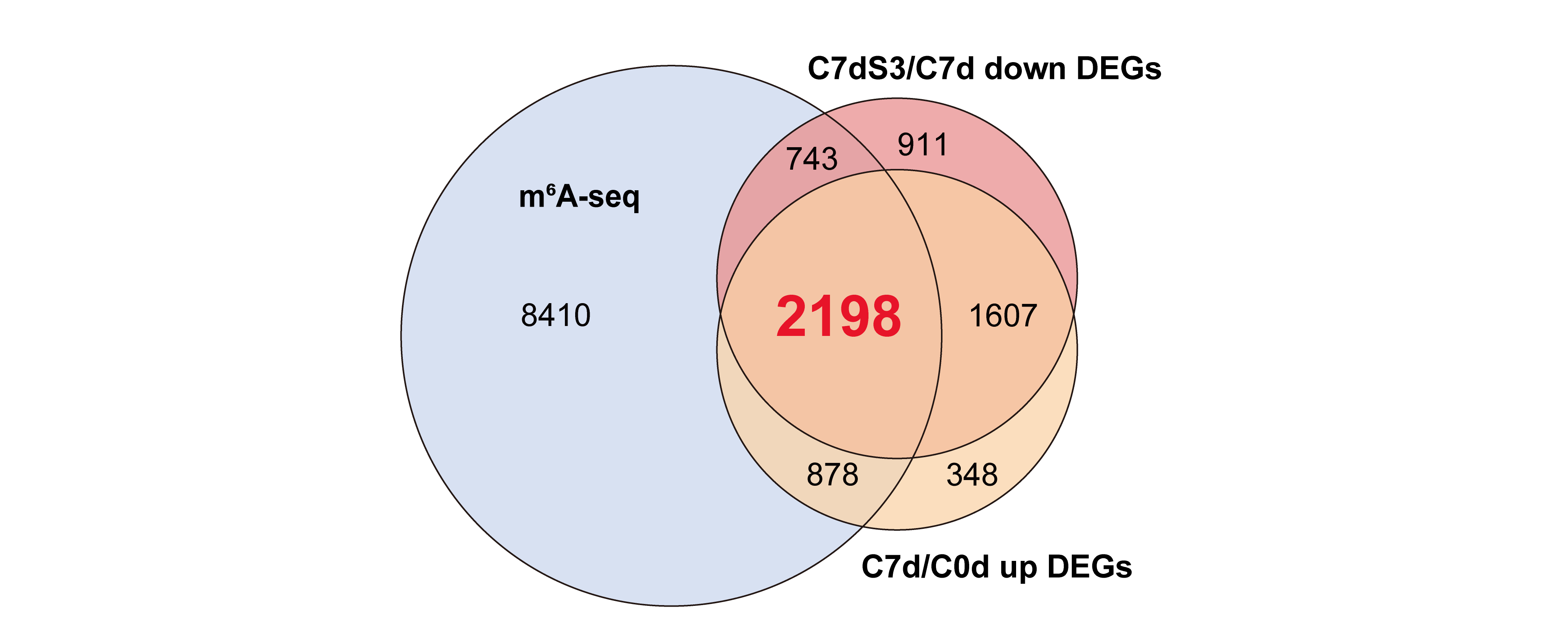


**Fig. S5** **Venn diagram showing the overlap genes of DEGs increased from C0d to C7d, DEGs decreased from C7d to C7dS3 and m^6^A genes.**


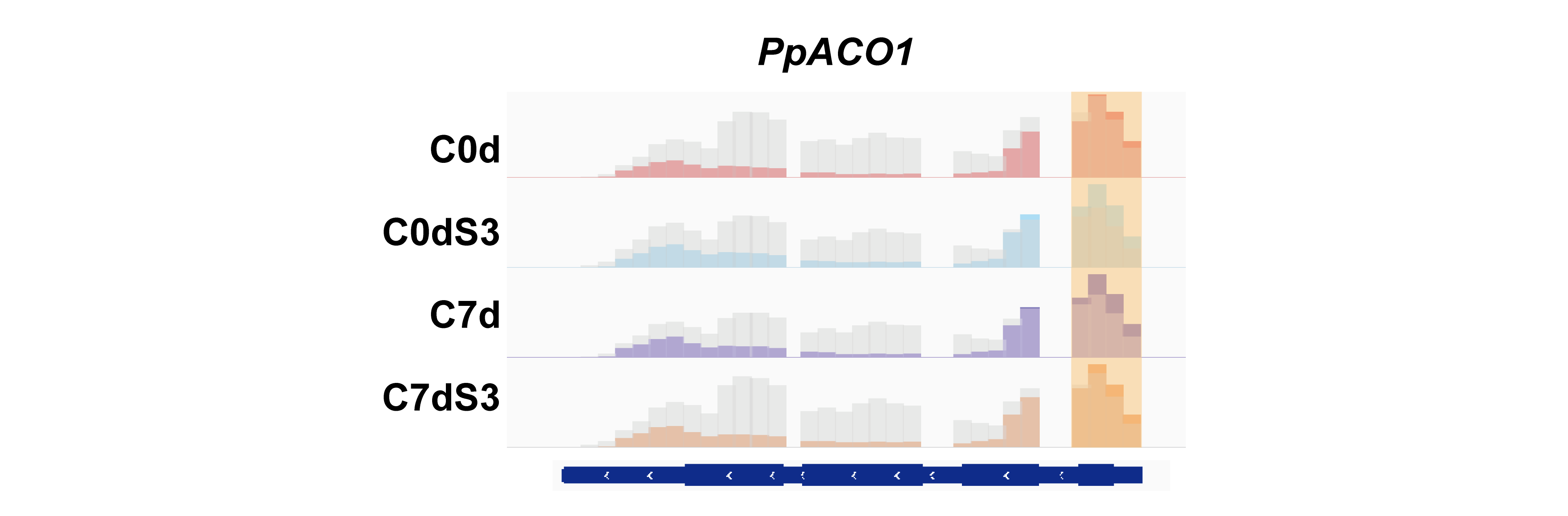


**Fig. S6** **m^6^A methylation status of *PpACO1* during storage were shown by Integrative Genomics Viewer (IGV).** The input reads are presented in the foreground. The ­yellow rectangle indicates the position of m^6^A targeted sites at 5' Untranslated Region (5’UTR).


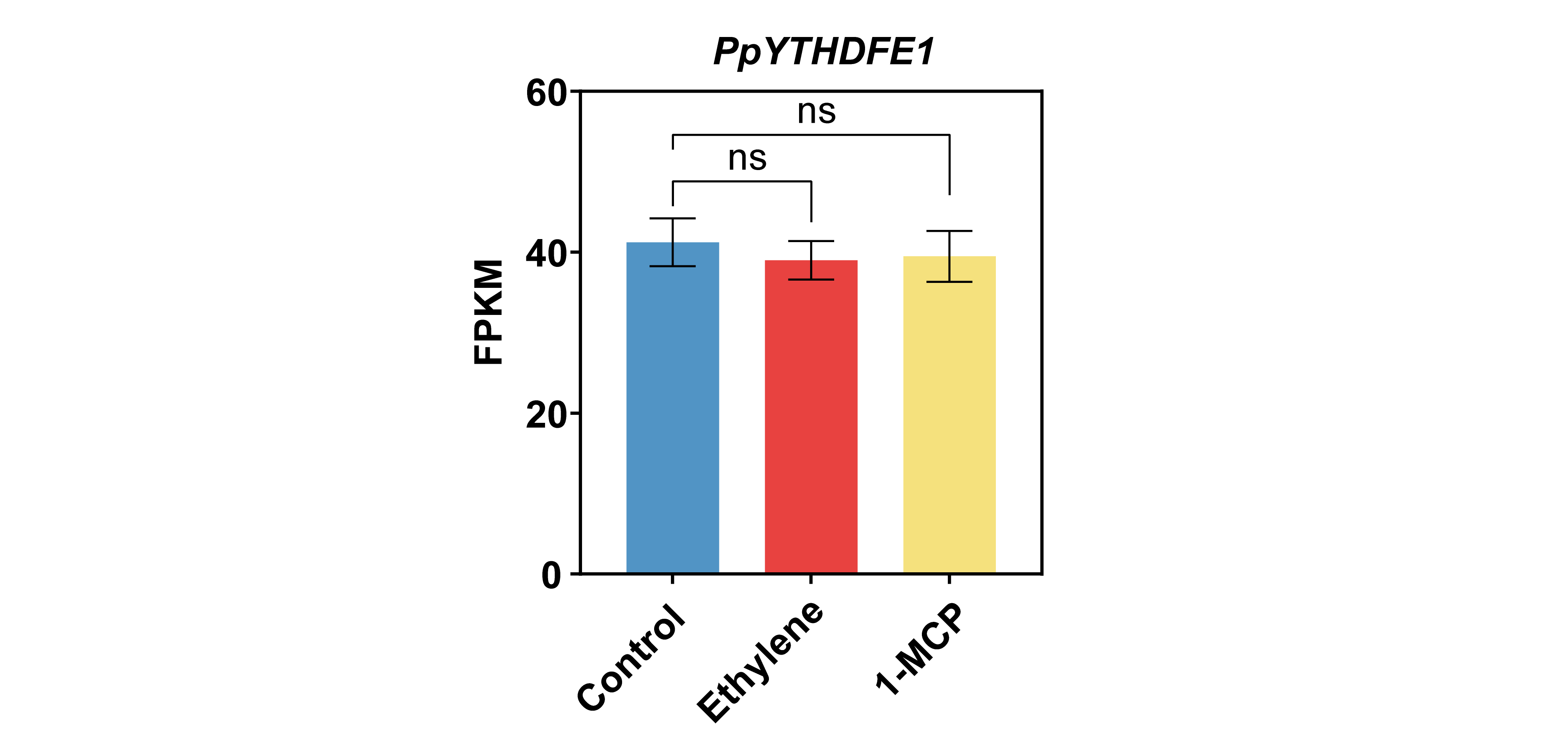


**Fig. S7** **Expression of *PpYTHDFE1* in peach fruit under ethylene and 1-MCP treatment for 72h.** (ns, no significant; Student’s *t* test). Data are presented as mean ± SD of three independent biological replicates.


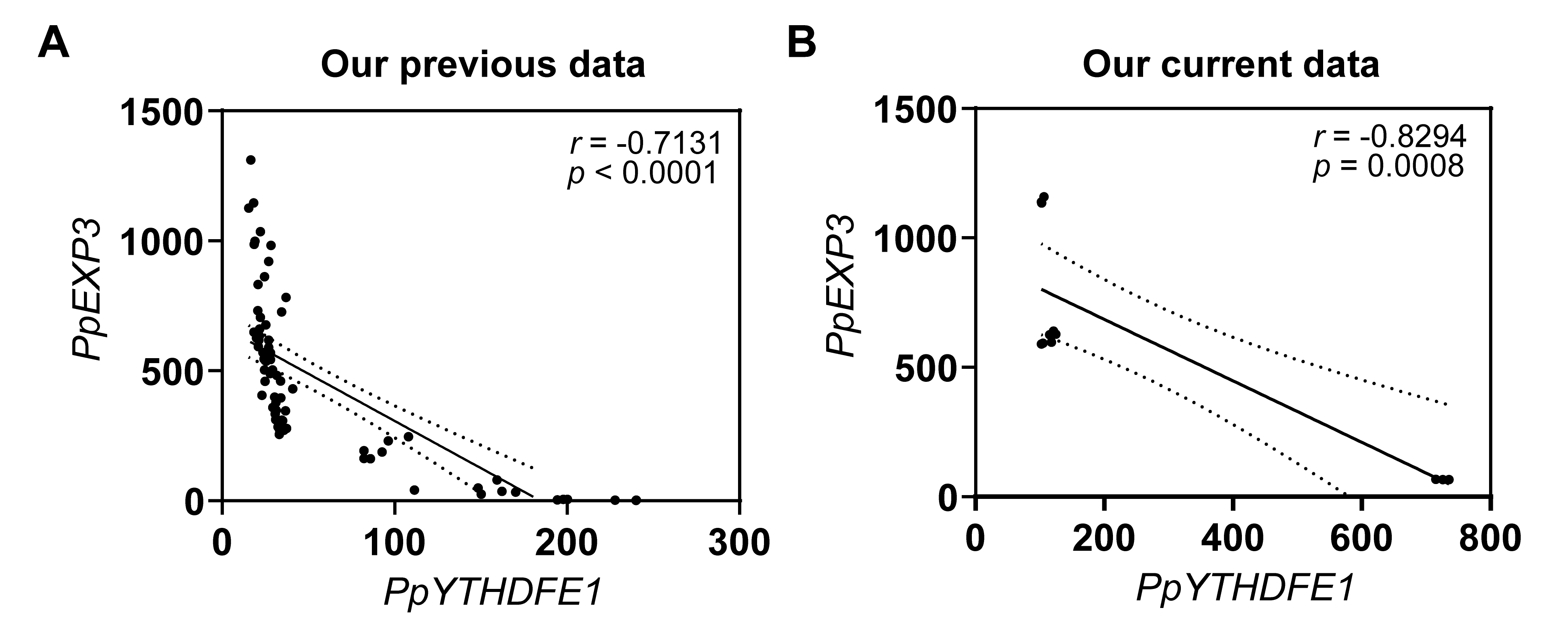


**Fig. S8** **Correlation analysis of the expression of *PpYTHDFE1* and *PpEXP3* during cold storage.** Our previous data (A) derived from 75 RNA-sequencing data and current data (B) derived from 12 RNA-sequencing data showing the negative correlation between P*pYTHDFE1* and *PpEXP3* expression. The solid line represents the linear regression line, and the dashed line represents the 95% confidence interval. The *r* value represents the Pearson correlation coefficient. The *p* value represents the significance of the correlation analysis results. Each point represents an independent sample.


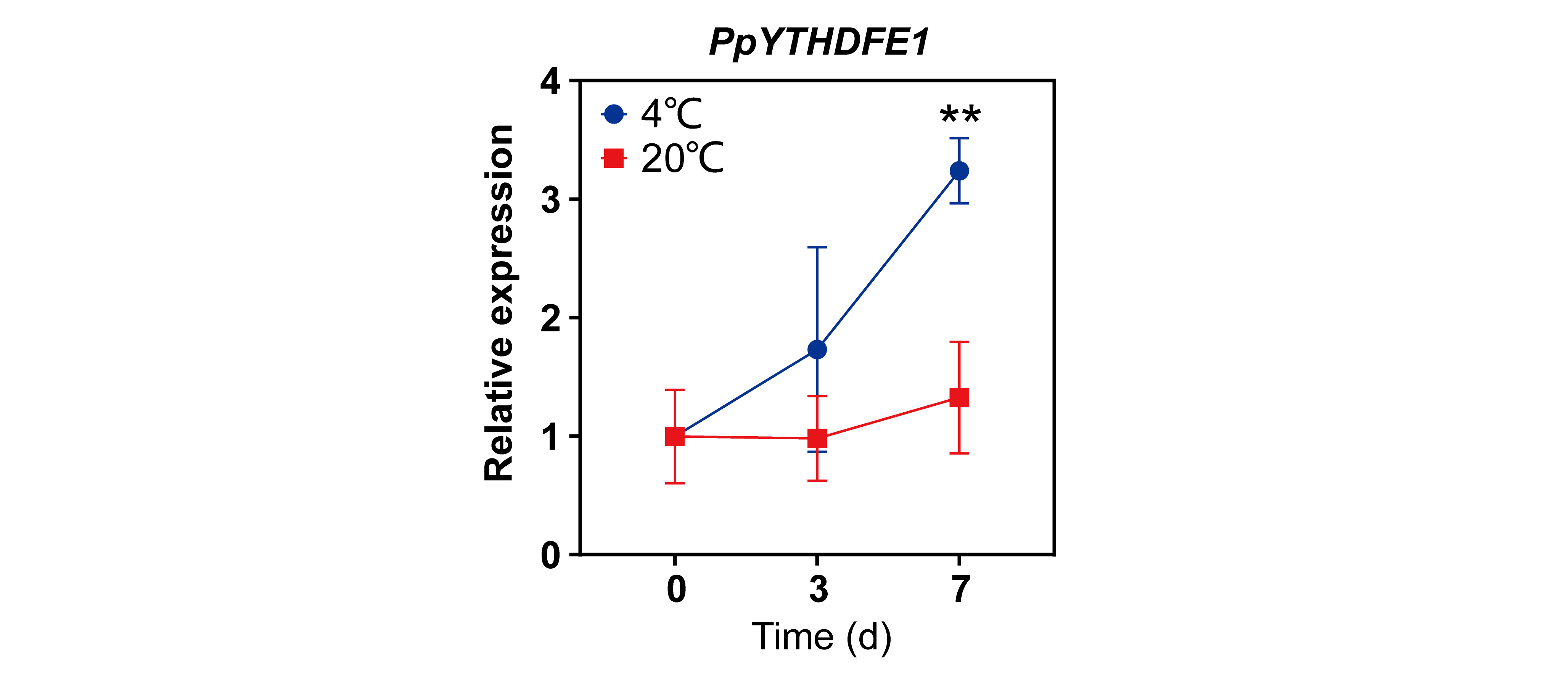


**Fig. S9** **Expression of *PpYTHDFE1* of peach flesh callus after storage at 4 ℃ or 20 ℃ for 3 and 7 days.** (**, P<0.01; Student’s *t* test). Data are presented as mean ± SD of three independent biological replicates. Data are presented as mean ± SD of three independent biological replicates.


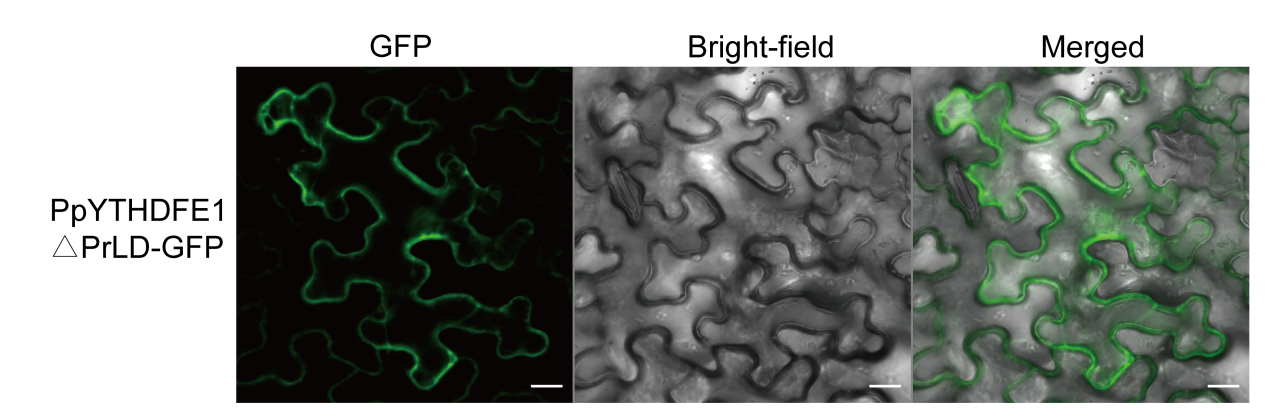


**Fig. S10** **PpYTHDFE1△PrLD‐GFP can not form liquid‐like cytosol condensates.**

Confocal images of PpYTHDFE1∆PrLD‐GFP subcellular localization in transgenic *Nicotiana benthamiana* leaves (Scale bar, 20 μm).


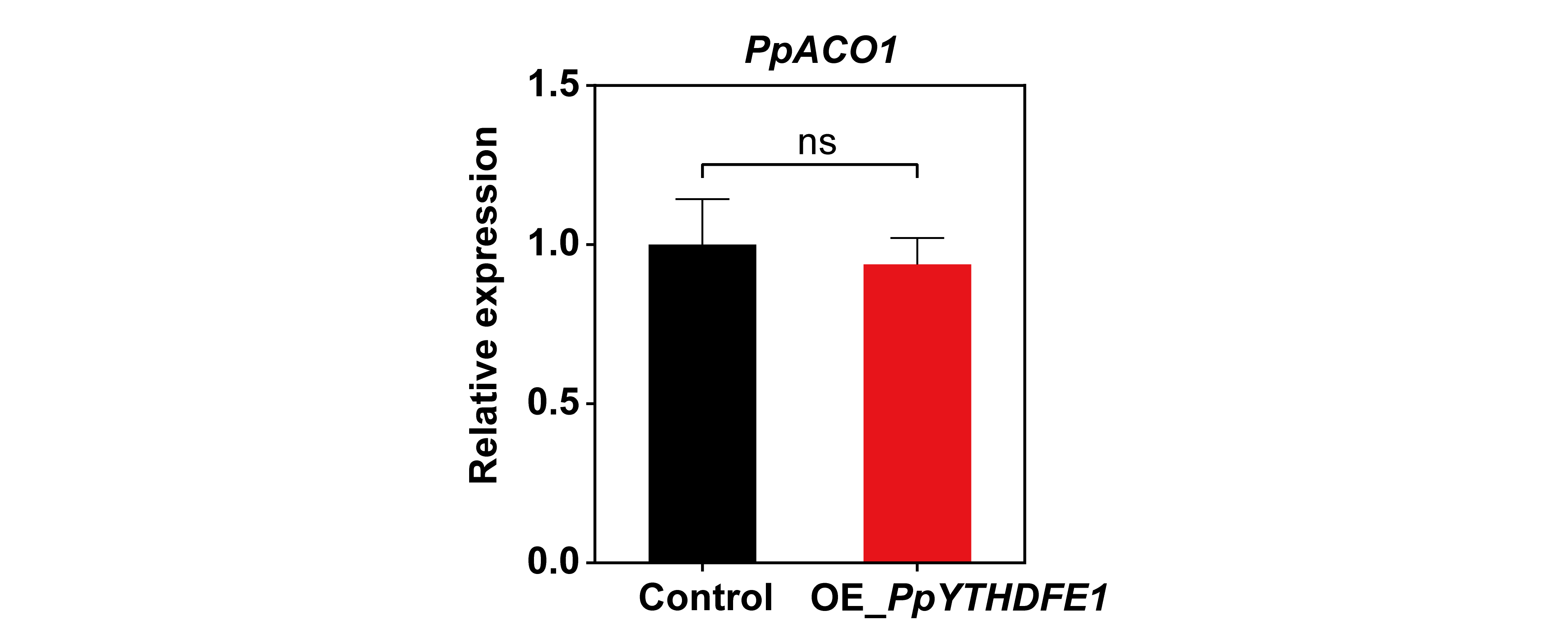


**Fig. S11** **Expression of *PpACO1* in peach flesh callus overexpressing *PpYTHDFE1*.** (ns, no significant; Student’s *t* test). Empty vectors serve as control. Data are presented as mean ± SD of three independent biological replicates.


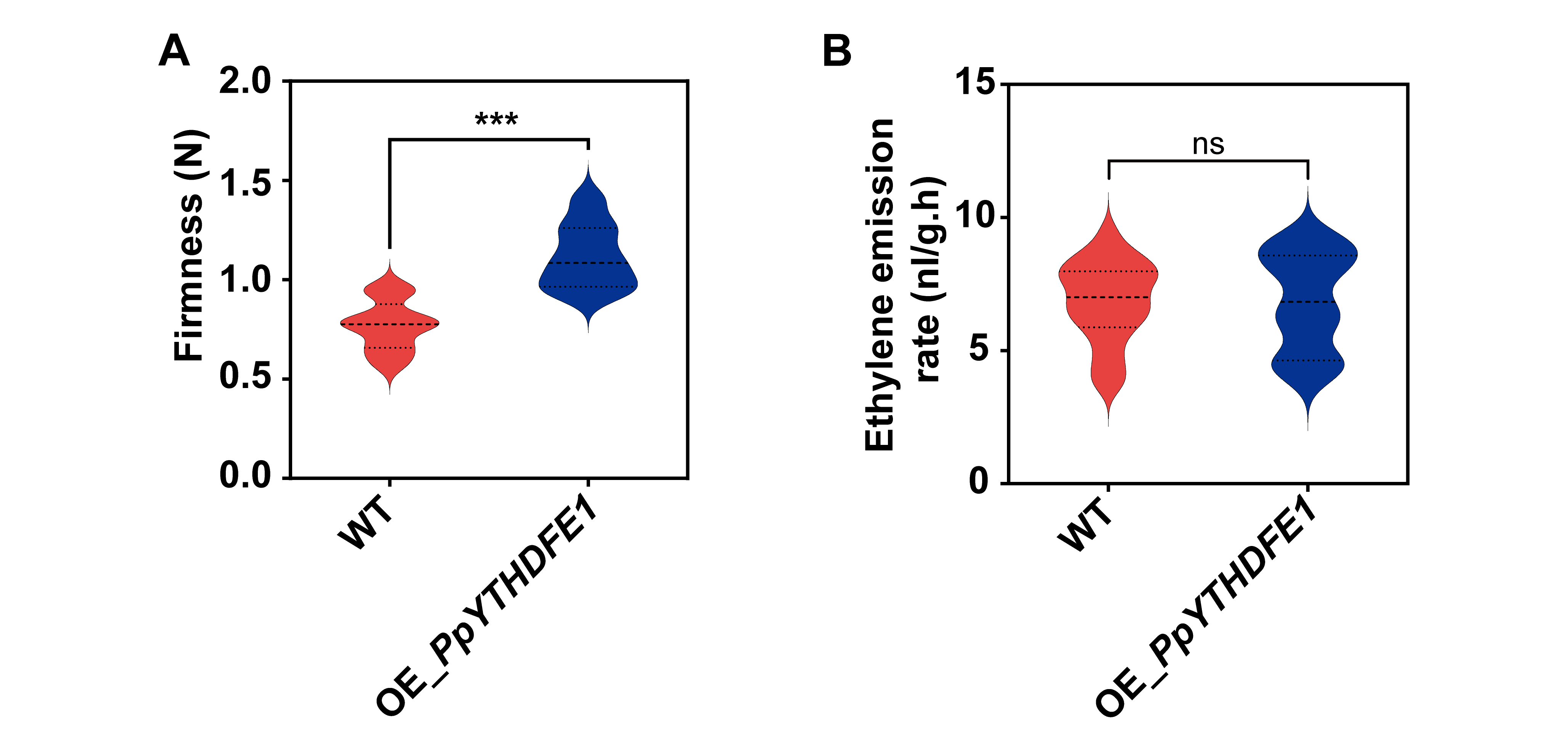


**Fig. S12** **Overexpressing *PpYTHDFE1* increases fruit firmness but not ethylene production.** (A) The violin chart displays the fruit firmness of wild type (WT) and transgenic *PpYTHDFE1* tomato (OE_*PpYTHDFE1*) results of nine independent experiments (***, P<0.001; Student’s *t* test). (B) The violin chart displays the fruit ethylene emission rate of wild type (WT) and transgenic *PpYTHDFE1* tomato (OE_*PpYTHDFE1*) results of nine independent experiments (ns, no significant; Student’s *t* test).


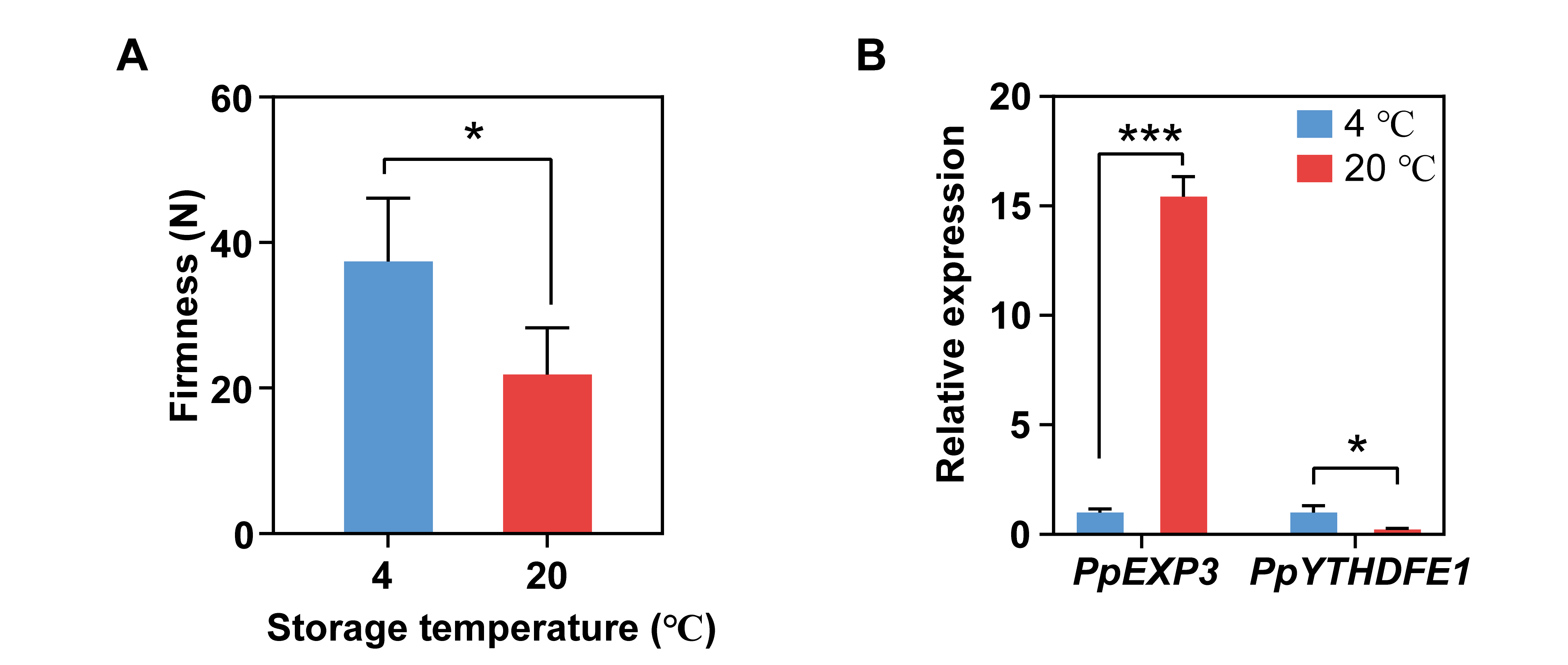


**Fig. S13** **Low temperature activation of *PpYTHDFE1* expression and inhibition of *PpEXP3* expression are also conserved in non-melting peach.** (A) Changes in firmness of peach fruits (“Qingzhoumi”) stored at 4 ℃ and 20 ℃ for 5 days (*, *P*<0.05; Student’s *t* test). Data are presented as mean ± SD (n = 9 biological replicates). (B) Changes in *PpYTHDFE1* and *PpEXP3* expression of peach fruits (“Qingzhoumi”) stored at 4 ℃ and 20 ℃ for 5 days (*, P<0.05, ***, P<0.001; Student’s *t* test). Data are presented as mean ± SD of three independent biological replicates.
